# Membraneless organelles in health and disease: exploring the molecular basis, physiological roles and pathological implications

**DOI:** 10.1038/s41392-024-02013-w

**Published:** 2024-11-18

**Authors:** Yangxin Li, Yuzhe Liu, Xi-Yong Yu, Yan Xu, Xiangbin Pan, Yi Sun, Yanli Wang, Yao-Hua Song, Zhenya Shen

**Affiliations:** 1 Department of Cardiovascular Surgery of the First Affiliated Hospital & Institute for Cardiovascular Science, State Key Laboratory of Radiation Medicine and Protection, Suzhou Medical College, Collaborative Innovation Center of Hematology, Soochow University, Suzhou, Jiangsu 215123 P. R. China; 2 Department of Orthopedics, The Second Hospital of Jilin University, Changchun, Jilin 130041 P. R. China; 3 NMPA Key Laboratory for Clinical Research and Evaluation of Drug for Thoracic Diseases, Key Laboratory of Molecular Target & Clinical Pharmacology and the State Key Laboratory of Respiratory Disease, School of Pharmaceutical Sciences, Guangzhou Medical University, Guangzhou, 511436 P. R. China; 4 Department of General Medicine, The Second Xiangya Hospital, Central South University, Changsha, Hunan 410011 P. R. China; 5 Department of Structural Heart Disease, National Center for Cardiovascular Disease, China & Fuwai Hospital, Chinese Academy of Medical Sciences & Peking Union Medical College, State key laboratory of cardiovascular disease, Beijing, 100037 P. R. China; 6 Department of Cardiovascular Surgery, Fuwai Yunnan Cardiovascular Hospital, Kunming, 650102 P. R. China; 7 Cyrus Tang Hematology Center, Collaborative Innovation Center of Hematology, Soochow University, National Clinical Research Center for Hematologic Diseases, The First Affiliated Hospital of Soochow University, State Key Laboratory of Radiation Medicine and Protection, Soochow University, Suzhou, 215123 P.R. China

**Keywords:** Cell biology, Molecular biology

## Abstract

Once considered unconventional cellular structures, membraneless organelles (MLOs), cellular substructures involved in biological processes or pathways under physiological conditions, have emerged as central players in cellular dynamics and function. MLOs can be formed through liquid-liquid phase separation (LLPS), resulting in the creation of condensates. From neurodegenerative disorders, cardiovascular diseases, aging, and metabolism to cancer, the influence of MLOs on human health and disease extends widely. This review discusses the underlying mechanisms of LLPS, the biophysical properties that drive MLO formation, and their implications for cellular function. We highlight recent advances in understanding how the physicochemical environment, molecular interactions, and post-translational modifications regulate LLPS and MLO dynamics. This review offers an overview of the discovery and current understanding of MLOs and biomolecular condensate in physiological conditions and diseases. This article aims to deliver the latest insights on MLOs and LLPS by analyzing current research, highlighting their critical role in cellular organization. The discussion also covers the role of membrane-associated condensates in cell signaling, including those involving T-cell receptors, stress granules linked to lysosomes, and biomolecular condensates within the Golgi apparatus. Additionally, the potential of targeting LLPS in clinical settings is explored, highlighting promising avenues for future research and therapeutic interventions.

## Introduction

**Fig. 1:**
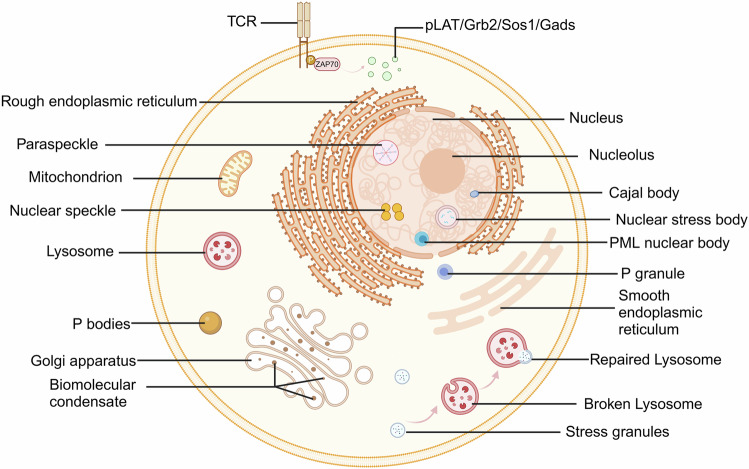
The localization of membraneless organelles. The cells contain membrane-bound organelles as well as MLOs such as Nucleolus, P granules, Paraspeckles, Stress Granules, Processing Bodies, Cajal Bodies, and Nuclear Speckles. Condensates associated with membranes include T-cell receptor, stress granules associated with lysosomes, and biomolecular condensates within the Golgi apparatus

Eukaryotic cells are equipped with two distinct types of organelles: membrane-bound organelles, which encompass the nucleus, endoplasmic reticulum, synaptic vesicles, mitochondria, lysosomes, etc., and MLOs like stress granules (SGs), processing bodies (P-bodies), nucleolus, and Cajal bodies (Fig. 1).^1,2^

Membrane-bound organelles facilitate organized biochemical reactions and regulatory processes and protect the cells by acting as barriers against harmful substances. For example, apoptosis can be triggered by releasing cytochrome c into the cytoplasm, while the release of nucleic acids activates innate immune pathways. In contrast, MLOs are cellular compartments lacking a surrounding lipid membrane yet exhibit distinctive organization within the cell. These structures are characterized by the dynamic assembly and disassembly of proteins and nucleic acids, creating specialized microenvironments. Unlike traditional membrane-bound organelles, most MLOs are characterized by their ability to undergo liquid-liquid phase separation (LLPS).^3–6^ MLOs engage in the exchange of various molecular substances with the surrounding environment, selectively concentrating specific substrates or enzymes to expedite specific biochemical reactions.^7^ Through phase separation, membraneless particles can temporarily store surplus biological macromolecules and even organelles, facilitating quick mobilization without the need for synthesis. An example of an MLO is the nucleolus, a distinct structure found in the nucleus. The nucleolus participates in ribosome biogenesis, with its assembly driven by the LLPS of nucleolar proteins and RNA molecules.^8^ Studies have shown that specific protein-protein and protein-RNA interactions govern the formation of nucleoli, highlighting the role of non-covalent interactions in the organization of MLOs.^9,10^ Another well-known MLO is the SGs, which form in response to cellular stress.^11^ Composed of RNA, proteins, and other biomolecules, SGs aid in the temporary storage of mRNA during stress conditions. This blocks the translation of unnecessary proteins, enabling the cell to prioritize its response to stress.^11,12^

The groundbreaking study conducted by Brangwynne et al. uncovered that cell function can be influenced through LLPS.^13^ Their research illustrated that the specification of germ cells involves the movement and condensation of P granules, which contain RNA and RNA-binding proteins (RBPs) and are evenly distributed in the unpolarized one-cell embryo. The relocation of P granules to the posterior half of the cell was linked to cell division. Notably, P granules exhibit liquid-like behavior, experiencing rapid dissolution/condensation and possessing the ability to fuse.^13^ Three years later, Rosen’s team established that small multivalent proteins can associate and form large gel-like complexes through LLPS.^14^ Within the same timeframe, McKnight’s group demonstrated that RNA granules are formed by ribonucleoproteins (RNPs) with LCR, facilitating a reversible process of phase transition.^15^

LLPS emerges as a possible mechanism involved in the formation of MLOs.^7^ This process entails the aggregation of molecules in a solution due to intermolecular interactions. Within living cells, only specific sets of proteins can undergo LLPS. Banani et al.^16^ proposed the subdivision of the membraneless particle component into scaffold and client. In the cellular context, a distinct group of biomolecules, known as scaffolds, can form condensates through multivalent interactions, typically involving repeating domains. Once the scaffold is established, it attracts other molecules, referred to as clients, which become bound to it. Certain proteins achieve LLPS through intrinsically disordered regions (IDRs),^17–21^ characterized by the presence of low-complexity sequence regions (LCRs) containing particular amino acids in high frequency (Table 1).^22–31^ One example is the prion-like LCRs,^32^ which exhibit chaperone-like functions that protect proteins from proteotoxic damage by regulating protein phase behavior.^33^ These LCRs are frequently found in proteins linked to neurodegenerative diseases, including amyotrophic lateral sclerosis (ALS).^34,35^ The distribution of LCR sequences is not random but enriched in RNA- and DNA-binding proteins associated with transcription and translation.^14^ The low-complexity domains (LCDs) are typically not found in isolation and are usually connected to folded domains, either as tails or internal linkers between folded domains.^36^ Multi-domain proteins that contain one or more folded domains along with linker and disordered regions are ideal candidates for modulating LLPS.^37^ Martin et al. studied the interaction between the folded and disordered domains of the RNA-binding protein hnRNPA1.^38^ They discovered that the folded RNA recognition motifs (RRMs) have a higher fraction of charged residues compared to the LCD. Electrostatic interactions between the folded domains and the LCD of hnRNPA1 contribute to phase separation at low ionic strengths.^38^ These interactions are disrupted at high ionic strengths, where the folded domains help to solubilize the LCD.^38^ Phase separation can also occur when the proline-rich motif (PRM) ligand binds to its Src homology 3 (SH3) domain.^39^ Both IDRs and SH3/PRM interactions are multivalent, meaning phase separation relies on interactions between multiple domains.^7^ Biogenesis of MLOs often begins with the nucleation of proteins or nucleic acids, which act as seeds for condensate formation.^40,41^ Nucleation can be facilitated by various factors,^42^ which may occur spontaneously or be triggered by specific cellular signals or stimuli.^43^ Once nucleation occurs, biomolecules undergo LLPS, resulting in the formation of a dense, coalesced phase within the cytoplasm or nucleoplasm.^44–46^ However, condensates do not need to be fluid, and not all condensates are MLOs.

**Table 1:** Database to predict LLPS formation

Database	Function	Website	Reference
D2P2	Prediction of disorder proteins	http://d2p2.pro	^22^
DrLLPS	Provide annotations of known and computationally detected LLPS-associated proteins, including IDR, post-translational modification, disease-associated information, etc.	http://llps.biocuckoo.cn/	^23^
LLPSDB	Provide protein sequence, modifications on specific amino acids, ability of coalescing with nucleic acid, phase behavior, experimental conditions	http://bio-comp.org.cn/llpsdb	^24^
MloDisDB	Provide information on LLPS and related diseases	http://mlodis.phasep.pro/	^25^
PhaSepDB	Provide annotation for phase separation (PS) entries, including the material states of the PS droplet, verification experiments, PS partners, mutations, modification, etc.	http://db.phasep.pro/	^26^
PhaSePred	Provides self-assembling and partner-dependent phase-separating protein prediction, and integrates scores from several PS-related predicting tools.	http://predict.phasep.pro	^27^
PhaSePro	Provides information on the biophysical driving force, biological function and regulation of LLPS.	https://phasepro.elte.hu	^28^
Pi-Pi predictor	Predicts LLPS formation in a given sequence based on **π-π** interaction.	10.7554/eLife.31486.021	^30^
PLAAC	Search protein sequences to identify the prion-like domains	http://plaac.wi.mit.edu/	^29^
PSPredictor	A sequence-based tool for the prediction of proteins with LLPS potential	http://www.pkumdl.cn/PSPredictor	^31^

In this review, we discuss the regulation of MLOs as a dynamic and multifaceted process that involves a combination of molecular interactions, signaling pathways, and cellular responses. We also discuss how dysregulation of these regulatory mechanisms can lead to aberrant condensate formation and contribute to various cellular dysfunctions and disease states.

## The mechanism and validation of LLPS

### The mechanism of LLPS

**Fig. 2:**
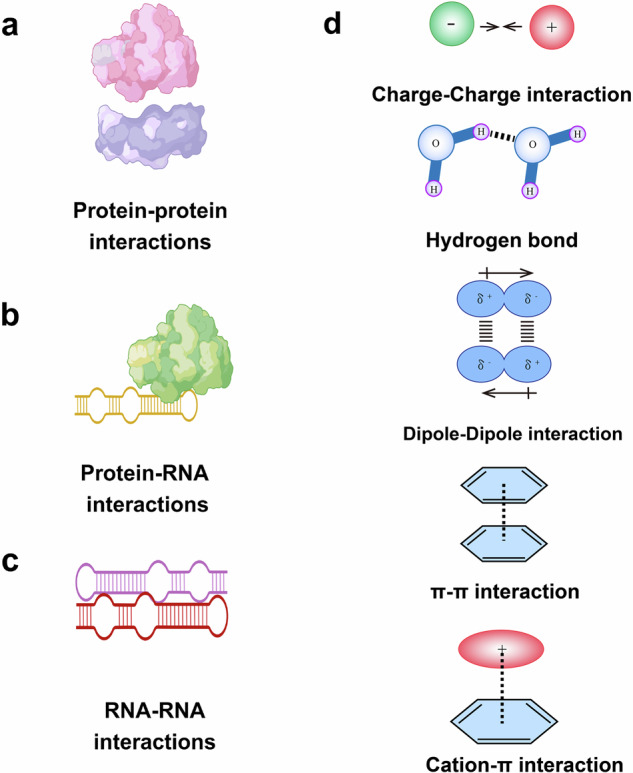
Multivalent interactions involved in phase separation. **a** Protein-protein interactions. **b** Protein-RNA interactions. **c** RNA-RNA interactions. **d** Multivalent interactions between IDRs include charge-charge interaction, hydrogen bond, Dipole-Dipole interaction, π–π stacking, and cation–π interaction

Phase separation is driven by weak, multivalent interactions between molecules, such as protein-protein interactions, protein-RNA interactions, or RNA-RNA interactions (Fig. 2).^47,48^ Following phase separation, the condensates undergo maturation and growth through the recruitment of additional biomolecules and the coalescence of small condensates into larger complexes.^49,50^ This process often involves specific interactions among proteins and RNAs that stabilize the condensate and contribute to its structural integrity. Maturation may also involve the incorporation of additional components to regulate condensate dynamics and function. MLOs exhibit dynamic properties, including fusion, fission, and exchange of components with the surrounding environment. Cellular signals or changes in the cellular environment can regulate the assembly, disassembly, or remodeling of MLOs in response to specific physiological cues or stress conditions. The dissolution of MLOs may occur through the reversal of phase separation, where the weak interactions holding the condensate together are disrupted.^51^ Dissolution can also be actively regulated by cellular processes, such as protein degradation, or by changes in cellular conditions that alter the stability or composition of the condensate.^52–54^ Overall, the biogenesis of MLOs is a highly dynamic and precisely regulated process that involves the self-assembly, phase separation, and maturation of biomolecules to form functional and dynamic structures within cells.

The stickers-and-spacers polymer framework describes the multivalent homotypic protein-protein and heterotypic protein-RNA interactions driving biomolecular condensation.^55–59^ In IDRs, residues that enable inter-chain attractive interactions include arginine (R) in R/G-rich IDRs and tyrosine (Y) in prion-like IDRs,^60^ commonly referred to as stickers. The linker residues connecting these stickers are known as spacers. The patterning of stickers and spacers can influence the physical properties of condensates and their phase behavior.^60,61^

The stickers are regions in the disordered protein that drive its compaction, making them crucial for the interactions leading to phase separation.^60^ Using nuclear magnetic resonance spectroscopy and small-angle X-ray scattering, aromatic residues were identified as the stickers in the protein hnRNAP1. These stickers alone are sufficient to explain phase behavior. Additionally, the arrangement of stickers (their patterning and the spacers between them) is necessary for functional LLPS. Clustering of stickers in the sequence led to the formation of solid aggregates rather than liquid droplets.^60^

**Fig. 3:**
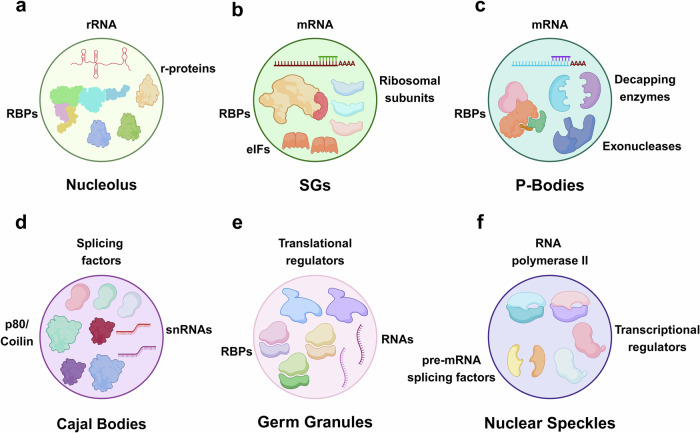
Molecular composition of various MLOs. **a** Nucleolus contains RBP: RNA-binding proteins (RBP), ribosomal RNA and proteins (rRNA and r-proteins). **b** SGs contain RBP, mRNA, ribosomal subunits, eIFs. **c** P-Bodies contain mRNA, decapping enzymes, RBP, exonucleases. **d** Cajal Bodies contain small nuclear RNAs (snRNAs), splicing factors and p80/coilin. **e** Germ Granules RNAs, RBPs, and translational regulators. **f** Nuclear Speckles contain pre-mRNA splicing factors, RNA polymerase II, and transcriptional regulators

The primary components of MLOs are proteins and nucleic acids, although other molecules such as lipids and metabolites may also contribute.^62–64^ These molecules interact via various non-covalent interactions, including electrostatic interactions, hydrogen bond, π–π, and dipole-dipole interactions (Fig. 2).^65^ RNA molecules, particularly long noncoding RNAs (lncRNAs) and ribosomal RNAs (rRNAs), are often found within MLOs (Fig. 3). RNA molecules can act as scaffolds or contribute to the material properties of the organelles.^66^ DNA can also be present, especially in the context of transcriptional condensates. MLOs serve diverse functions within cells, including the spatial and temporal organization of biological processes such as responses to stress, transcription and translation.^7,67,68^ By concentrating reactants and enzymes, MLOs can accelerate reaction rates and regulate reaction specificity. MLOs help spatially organize cellular components and play crucial roles in oxidative stress, heat shock, and nutrient deficiency.^69^ They can dynamically assemble to sequester and protect essential molecules, prevent the aggregation of misfolded proteins or facilitate the degradation of damaged components. Certain MLOs, such as transcriptional condensates, facilitate gene expression.^70^ They concentrate transcription factors, RNA polymerases, and regulatory RNAs to control the transcription of specific genes in response to developmental cues or environmental signals (Fig. 3).^51,71,72^ By bringing together signaling molecules and effectors, they facilitate the efficient propagation and regulation of cellular signals.^73^

RNA molecules, including lncRNAs and specific mRNAs, can regulate the formation and properties of MLOs.^74^ RNA molecules can act as scaffolds, regulators, or structural components within condensates, influencing their assembly and function.^75^ RNA regulation modulates the composition, stability, and activity of MLOs, impacting processes such as RNA metabolism, translation, and RNA-based regulation of gene expression.

Conceptualizing the dynamic assembly of mRNAs into RNA granules has been challenging. To address this issue, Han et al. simulated the formation of these granules by treating mouse brain extracts and human cell lysates with biotinylated isoxazole (b-isox).^76^ Deep sequencing of the associated RNAs revealed an enrichment of mRNAs known to be recruited to neuronal granules, which are used for dendritic transport and localized translation at synapses. The precipitated mRNAs contained extended 3′UTR sequences and were enriched in binding sites for known granule-associated proteins. The mRNAs enriched by b-isox precipitation had an average 3′UTR length roughly five times longer than those excluded from the precipitate. They demonstrated that hydrogels composed of the low-complexity (LC) sequence domain of FUS recruited and retained the same mRNAs selectively precipitated by the b-isox chemical. Interestingly, phosphorylation of the LC domain of FUS prevented hydrogel retention.

Protein-protein interactions and the composition of MLOs are tightly regulated by specific factors, including chaperones, binding partners, and regulatory proteins. Competitive binding and allosteric regulation influence the recruitment and retention of proteins within condensates.^77,78^ Regulation of protein interactions and composition modulates the structural integrity, function, and adaptability of MLOs, influencing cellular processes such as metabolism, signaling, and stress responses.

Cellular signals and stress conditions regulate the formation, dissolution, or remodeling of MLOs in response to specific physiological cues. Signaling pathways, such as those mediated by kinases, phosphatases, and transcription factors, can directly modulate the properties of condensates.^79,80^ Regulation by cellular signaling and stress responses allows MLOs to dynamically adapt to changing cellular environments, modulating processes such as gene expression, SG formation, and cellular homeostasis.

Molecular chaperones and quality control mechanisms regulate the maturation, stability, and turnover of proteins within MLOs. Chaperones facilitate protein folding, prevent protein aggregation, and promote the degradation of misfolded or damaged proteins within condensates.^81,82^ Chaperone-mediated regulation ensures the proper assembly, function, and maintenance of MLOs, preventing aberrant condensate formation and maintaining cellular proteostasis.

Using passive microrheology with optical tweezers (pMOT), Alshareedah et al. quantified the viscoelastic properties of a series of artificial condensates formed by disordered sticker-spacer polypeptides and RNA.^83^ They demonstrated that at shorter timescales, peptide-RNA condensates exhibit an elastically dominant rheological response, whereas at longer timescales, these condensates behave as predominantly viscous liquids.^84^ The network relaxation time, or the timescale at which the condensate transitions from an elastically dominant to a viscous behavior, is determined by the chemical identities of the sticker and spacer residues in the polypeptide chain.^84^ Additionally, they showed that the viscous and elastic regimes of these condensates can be tuned by the sequences of the polypeptides and RNA, as well as their mixture compositions.^84^

It is theoretically conceivable that the fluidity and stiffness of a network can be encoded by the sequence composition and sticker identity in a polypeptide chain.^85^ Recent studies have shown that biomolecular condensates are network fluids with variable viscoelastic properties.^10,45,86,87^ This viscoelasticity is likely due to transient network-like structures formed through physical crosslinking among protein and/or RNA chains with finite bond lifetimes.^45^ Consequently, there is growing interest in using experimental methods to probe the material properties of condensates across different timescales.

RNA-binding proteins such as FUS, TDP43, and hnRNPA1 form dynamic liquid-like condensates that can transition to a solid state over time, a process known as maturation or aging of condensates. This transition can result in pathological aggregates.^34,35,88,89^ It is now widely accepted that this transition is associated with the viscoelastic properties of these proteins.^90–92^

Traffic into and out of the nucleus occurs through nuclear pore complexes that span the two membrane bilayers of the nuclear envelope. Small molecules and ions passively diffuse through these pores, while larger molecules are restricted from entry. The transport of large macromolecules requires binding to nuclear transport receptors (NTRs). The nuclear pore complex is mainly composed of proteins known as nucleoporins (Nups), some of which contain IDRs. These disordered proteins, called FG nucleoporins (FG-Nups), have multiple phenylalanine–glycine repeats (FG repeats) in their amino acid sequences.^93^ It is now understood that mixtures of FG-domains and NTRs undergo phase separation to facilitate passage of NTR carried cargos through the nuclear pore. This process involves a competitive disruption of adjacent inter-repeat contacts, transiently opening adjoining meshes.^94^ NTRs may act as crosslinkers between FG-domains, converting them into elastic and reversible hydrogels.

There are also condensates that are membrane-associated, including T-cell receptor, stress granules associated with lysosomes, and biomolecular condensates within the Golgi apparatus (Fig. 1). Activation of various cell surface receptors leads to the reorganization of downstream signaling molecules into micrometer- or submicrometer-sized clusters. The functional implications of such clustering have remained unclear. Su et al. biochemically reconstituted a 12-component signaling pathway on model membranes, starting with T-cell receptor (TCR) activation and culminating in actin assembly. Upon TCR phosphorylation, downstream signaling proteins spontaneously formed liquid-like clusters that enhanced signaling outputs both in vitro and in human Jurkat T cells. These reconstituted clusters were rich in kinases but excluded phosphatases, thereby promoting actin filament assembly by recruiting and organizing actin regulators. These findings illustrate that protein phase separation can create distinct physical and biochemical compartments that facilitate signaling.^95^

Bussi et al. explored mechanisms of lysosomal membrane repair. Using super-resolution microscopy on human stem cell-derived macrophages, they observed that chemically induced lysosomal rupture led to the formation of G3BP-positive granules in a plug-like pattern at the sites of membrane damage. Their findings revealed that stress granules nucleate near damaged endolysosomes, acting as protective plugs that stabilize the ruptured membrane and facilitate efficient repair.^96^

Extensive studies have revealed a wide variety of nuclear and cytosolic MLOs. However, there is a growing interest in protein condensates associated with membranes of the secretory pathway, such as the endoplasmic reticulum and the Golgi apparatus. The Golgi apparatus is essential for protein sorting and lipid metabolism, characterized by its stacked, flattened cisternal structure and distinct polarity with cis- and trans-faces that coordinate various protein maturation and transport processes. Central to its structural integrity and organization are the Golgi Matrix Proteins (GMPs), mainly composed of Golgins and GRASPs. These proteins contribute to the unique stacked and polarized structure of the Golgi, ensuring the precise localization of Golgi-resident enzymes crucial for accurate protein processing. Research has shown that GMPs across different eukaryotic lineages have a significant tendency to form biomolecular condensates. Rebane et al. demonstrated that GM130, a member of the Golgin family, can form droplets with internal component mobility in vitro.^97^ Furthermore, when overexpressed in cells, GM130 exhibited dynamic condensates in the nucleus.^15^ Using optical and fluorescence microscopy, Mendes et al. observed the formation of protein-rich, round-shaped condensates of GRASP55.^98^

Neuronal transmission depends on the regulated release of neurotransmitters, which are stored in synaptic vesicles (SVs). These SVs are highly mobile, allowing them to be quickly recruited to the plasma membrane for rapid release during neuronal activity. At synapses, SVs form tight clusters, acting as a reservoir from which they are drawn for exocytosis during sustained activity. Synapsin, a family of proteins, is a major component of the matrix connecting SVs and has long been implicated in regulating neurotransmitter release at synapses. Milovanovic et al. discovered that synapsin can form a distinct liquid phase in an aqueous environment.^99^ Hoffmann et al., using two-color SMT and super-resolution imaging in living axons, demonstrated that synapsin 1 drives the accumulation of SVs in boutons. They found that synapsin 1 condensation is sufficient to ensure the reliable confinement and motility of SVs in vivo.^100^

Golgins are a plentiful class of peripheral membrane proteins found in the Golgi apparatus. Rebane et al. demonstrated that overexpression of GM130, the most abundant Golgin at the cis Golgi, leads to the formation of liquid droplets in cells. This behavior is similar to that observed in many intrinsically disordered proteins with low-complexity sequences, even though GM130 itself is neither low in complexity nor intrinsically disordered.^97^ A subsequent study by the same group revealed that other members of the Golgin family, including golgin160, GMAP210, golgin97, golgin245, GCC88, and GCC185, also form condensates when overexpressed.^101^

A prominent feature in the oocytes of many diverse organisms is the Balbiani body (Bb).^102^ The Bb is a non-membrane-bound compartment that, in addition to localized RNAs and proteins, contains a high number of membrane-bound organelles such as mitochondria and endoplasmic reticulum. Velo1 has been identified as the most enriched protein in the Xenopus Bb that is not part of the membranous organelles. When Velo1-GFP is injected into oocytes, it localizes to Balbiani bodies and fills the gaps between mitochondria. Velo1 forms a stable matrix in the Bb, as evidenced by its very slow turnover after photobleaching.^86^ These observations suggest that Velo1 acts as a structural glue, holding the organelles together in the Balbiani body.^103^ How can a protein act as a glue to bring organelles together in a stable yet reversible matrix in the cytoplasm? Velo1 is a highly disordered protein with a prion-like domain (PLD) in its N-terminus and a positively charged C-terminus that binds to RNA. In vivo and in vitro experiments have demonstrated that Velo1 is a physiological amyloid that forms cages around organelles.^103^

MLOs have a distinct organization of proteins at their interfaces, which regulate their interactions with membranes.^104^ Using graphene-based sensors, Hoffmann et al. discovered that synapsin condensates generate strong electrical responses, which are absent when synapsin is present in a single phase.^105^ These experiments suggest that synapsin/synaptic vesicle condensates could act as charge centers at synaptic boutons, adding a new layer of regulation to neurotransmission.^105^ Additionally, Dai et al. demonstrated that the interface of condensates can drive spontaneous redox reactions both in vitro and in living cells.^106^

Although LLPS is believed to be the main mechanism behind the formation of many MLOs, some MLOs, like glycogen granules, do not rely on LLPS because they are not liquid, which prevents them from readily exchanging their contents with the environment.^83^ Glycogen granules serve as energy reservoirs that can be mobilized when needed.^83^

### Validation methods

Disordered regions can be identified using predictive algorithms such as PLAAC.^29^ The prediction can then be tested experimentally by reconstitution of LLPS with minimal components and verified using mutants.^107^ The formation of LLPS is characterized by the appearance of spherical droplets, which can be visualized under a microscope. LLPS can also be detected via turbidity measurement through either optical density or direct static light scattering. The material state of condensates can be monitored by measuring the ratio of the viscosity to surface tension using fluorescence or transmitted light microscopy.^1^

An optogenetic platform was developed to track the formation of the droplet in vivo using blue light-activated IDR-Cry2 fusion protein.^108^ To achieve this, the researchers combined the “sticky” IDR from different proteins to the photolyase homology region (PHR) of Arabidopsis thaliana Cry2, which interacts autonomously when exposed to blue light.^108^ The researchers demonstrated that by activating light-sensitive proteins, they could induce phase transitions and form MLOs.^109^ These transitions could be reversed simply by turning off the light. Through enhancing light intensity and protein levels, the investigators achieved greater control over the transitions, allowing them to dictate the formation of condensed liquid protein droplets and solid-like protein aggregates, which may be associated with diseases.^108^

The liquidity of droplets can be measured quantitatively in living cells using fluorescence recovery after photobleaching (FRAP).^1,110^ Considering the difficulties in validating phase separation in vivo, the LLPS phenomenon may have been over-interpreted in the literature. As pointed out by McSweiggen et al., many reported LLPS studies were descriptive rather than quantitative. According to their criterion, a study reporting the roundness of the droplet is considered qualitative. In contrast, a study that measures the degree of roundness is considered quantitative.^110^ It is important to note that not all proposed MLOs adhere to these criteria.

## MLOs and biomolecule condensates formed by LLPS

MLOs, also known as biomolecular condensates, encompass a diverse array of structures found within cells. These condensates form through LLPS, driven by the self-assembly of specific proteins and nucleic acids. MLOs encompass a diverse array of structures found within cells, containing specific components.

### Ribosomes

Ribosomes are essential macromolecular machines found in all cells, responsible for translating messenger RNA (mRNA) into proteins. They link amino acids together in the order specified by mRNA codons to form polypeptide chains. Ribosomes consist of two primary subunits: the small and large ribosomal subunits, each containing one or more rRNA molecules and numerous ribosomal proteins (r-proteins). Collectively, ribosomes and their associated molecules are known as the translational apparatus. Unlike many other organelles, ribosomes are not membrane-bound; they are MLOs that float freely in the cytoplasm. The lack of a membrane is crucial as it facilitates the efficient transport of newly synthesized proteins, reducing the energy required for this process.

### Nucleolus

Nucleolus is located within the nucleus, enriched in rRNA, ribosomal proteins, and RNA-binding proteins (RBPs) such as RBM28 (RNA-binding motif protein 28).^54,111^ Its function is involved in ribosome biogenesis, where it serves as the site of rRNA transcription, processing, and assembly of ribosomal subunits.^112,113^ The nucleolus is the largest MLO driven by LLPS. Experiments using FRAP technique and nucleolar proteins have demonstrated a significant level of exchange between these proteins and the surrounding nucleoplasm.^114^ Many nucleolar proteins contain IDRs, often with charged domains, which are crucial for driving phase separation. It is known that nucleolar proteins such as fibrillarin and nucleolin possess Gly–Arg-rich (GAR) domains.^115^ As nascent transcripts come out from the fibrillar center (FC)–dense fibrillar component (DFC) interface, they bind to the RNA-binding domain of fibrillarin (FBL). FBL interacts with itself via its GAR domain, which is composed of IDRs. This self-association facilitates the formation of the DFC phase and initiates the processing of pre-rRNA.^116^

### P granules

P granules are RNA granules that can be found in the perinuclear area of the germ cells of C. elegans. During most of germline development, P granules are perinuclear, but they become cytoplasmic during the transition from oocyte to embryo.^117^ The defining constituents of P granules include two classes of RBPs: the RGG-domain proteins PGL-1 and PGL-3, and the DEAD-box proteins GLH1-4.^118^ P granules were the first cytoplasmic RNA granules identified to exhibit liquid-like property.^95^ They are roughly spherical, can fuse with one another, and exchange constituents with the cytoplasm. The spontaneous LLPS of PGL-3 with RNA drives P granule formation. However, the PGL-3 phase is unstable and needs a second phase for stabilization in embryos. This second phase is created by congregations of the disordered protein MEG-3, which is associated with PGL-3 liquid droplets in the embryo’s posterior. The congregations of these gel and liquid phases provide regional stability and dynamic properties essential for localized P granule assembly.^119^

### Paraspeckles

Paraspeckles are nuclear bodies involved in gene expression regulation in mammalian cells, though their exact function is not yet fully understood. They are believed to control gene expression by segregating proteins or mRNAs with inverted repeats in their 3′ UTRs.^109,120^ Paraspeckles are protein-rich nuclear organelles constructed around a specific long noncoding RNA (lncRNA) scaffold. Initially identified in 2002 as nuclear foci containing paraspeckle component 1 (PSPC1),^121^ paraspeckles are now recognized to contain at least three RBPs such as PSP1/2 (paraspeckle protein 1/2), p54/nrb and nuclear paraspeckle assembly transcript 1 (NEAT1), which is an architectural lncRNA that attracts proteins containing LCDs to initiate the LLPS process to form paraspeckles.

### Promyelocytic leukemia protein nuclear bodies (PML NBs)

PML NBs are macromolecular multi-protein complexes exhibiting properties of phase-separated liquid-like droplets, undergoing LLPS through heterotypic multivalent interactions between proteins and RNA molecules.^122,123^ The participation of LLPS in the formation of PML NBs has been confirmed by FRAP.^124^ PML NBs were first observed under electron microscopy, and their importance was realized due to their reversible disruption by treatment in a rare form of leukemia.^125^ These bodies recruit multiple partner proteins and have emerged as sumoylation factories responsive to interferon and oxidative stress. The composition of PML NBs is controlled by PML sumoylation and mRNA concentration.^122^ PML NBs mediate interferon-induced viral constraint and implement stress-induced senescence.^126^ The PML protein (TRIM19) is the crucial component of PML NBs. It contains the Ring finger, B-box, and coiled-coil domains that function as a scaffold protein necessary for the assembly of these bodies.^127^ Other proteins that reside in PML NBs are termed client proteins. One such client protein is TRIM33, which mediates nodal signaling in mouse embryonic stem cells.^128^ Malfunction of PML NBs often leads to acute leukemia and other severe diseases. The molecular basis of arsenic’s success in treating acute promyelocytic leukemia (APL) lies in rescuing PML NBs. Compared to wild-type PML NBs, the PML A216V variant from arsenic-resistant leukemia patients significantly impairs LLPS.^124^

### Nuclear stress bodies (nSBs)

nSBs were initially recognized as the primary site of heat-shock factor-1 (HSF1) buildup in stressed cells.^129^ These structures are typically located close to the nucleoli. Like nuclear speckles,^130^ nSBs are rapidly evolving, as evidenced by the swift and easily reversible movement of HSF1.^131^ This dynamic nature challenges the notion that nSBs are merely clusters of misfolded proteins and suggests an involvement in cell recovery from stress. The formation of nSBs necessitates the engagement of HSF1, which modulates the transcription factor’s transactivating capacity.^132^ Originally believed to be accumulations of denatured proteins, this perspective shifted when the Morimoto’s group demonstrated that the formation of nSB can be triggered by various stressors, not all of which cause the denaturation of proteins.^133^ The amount of stress bodies correlates with cell ploidy;^134^ for instance, up to ten nSBs can be observed in cells that acquired infinite growth, while only two can be observed in primary cells. Notably, nSBs can be found in all primate cells but not in rodents.^135^ This observation suggests that the noncoding regions of the genome poorly conserved among different species might be involved in the formation of nSB.^135^ Under stress conditions. nSBs regulate gene expression by altering chromatin structure and attracting transcription and splicing factors. The nSB proteome includes at least 133 proteins, with over 90% being highly disordered and 66% having a high probability of promoting LLPS.^136^

### Amyloid bodies (A-bodies)

The aggregation of amyloid can occur under physiological conditions.^137^ The A-bodies share the same biophysical characteristics as plaques associated with various amyloid-related diseases. The formation of A-bodies under stress conditions allows cells to gather enough proteins before entering an inactive state. It is proposed that cells can convert native proteins into an amyloid-like solid phase in a reversible process involving post-translational pathway. Different subtypes of A-bodies with discrete protein compositions can be formed in response to different stimuli. The formation of A-bodies may have evolved as a highly specialized mechanism for enduring various stressors.^138^

### Stress granules (SGs)

SGs are located in the cytoplasm and are formed in response to various stresses, such as heat, over-production of free radicals, oxygen or nutrient deprivation. It contains mRNAs, RBPs (e.g., TIA-1, G3BP), translation initiation factors, and ribosomal subunits.^96,139^ SGs sequester and store untranslated mRNAs during stress conditions, allowing cells to prioritize essential processes and protect mRNAs from degradation.^78^ SGs contain non-translating messenger ribonucleoproteins (mRNPs) that are formed when mRNAs stall during the initiation phase of translation, either due to drug treatments or stress responses.^140^ SGs are fluid architectures, exhibiting quick turnover of components, dissociation into translating mRNPs, and removal through autophagy. The current paradigm suggests that various RNP granules are formed through LLPS and directed by the interplay between IDRs.^16,17,33,141,142^

SG formation can impact cellular reactions in two main ways. First, the increased amount of components within stress granules shifts the equilibrium of interacting molecules toward linked states. For instance, amidst a viral infection, SGs enhance the innate immune response by engaging and activating antiviral proteins such as OAS, RIG-1, RnaseL and PKR. Second, SGs can regulate signaling pathways by trapping components from the bulk cytosol, thereby limiting their interactions. This sequestration can affect pathways involving TOR, RACK1, or TRAF2.^143^ Mutations that influence the establishment or maintenance of SGs are linked to myopathies, ALS, and frontotemporal lobar degeneration (FTLD).^144,145^ Additionally, stress granules are implicated in both cancer progression and management, with many chemotherapeutic agents promoting their formation.^146,147^

### Processing bodies (P-Bodies)

P-bodies are distinct cytoplasmic foci in eukaryotic cells, formed by phase separation and comprising decapping factors Dcp1 and Dcp2 and other enzymes that break down mRNAs in the 5′ to 3′ direction.^148^ These highly conserved structures are found in somatic cells of vertebrates, invertebrates, plants, and yeast. P-bodies play essential roles in several RNA-related processes, including general mRNA degradation and microRNA (miRNA)-induced mRNA suppression.^149^ They serve as sites for mRNA storage, degradation, and surveillance, playing a role in regulating gene expression and mRNA quality control.^150^

### Cajal bodies (CBs)

CBs are unique sub-nuclear structures found in eukaryotic cells, typically located in the nucleus near the nucleolus. They contain components involved in RNA processing and modification, including splicing factors and small nuclear ribonucleoproteins (snRNPs). CBs participate in snRNP biogenesis and the assembly of the spliceosome, which is involved in the splicing of pre-mRNA.^54^ They are crucial for RNA metabolism and the assembly of RNPs involved in processes such as telomere maintenance, splicing, transcription, and ribosome biogenesis.

### Germ granules

Germ granules are specialized structures enriched in RNAs found exclusively in the cytoplasm of germ cells (e.g., oocytes, spermatocytes). These granules house essential factors for germ cell development and likely serve as central hubs for the posttranscriptional regulation of gene expression. It contains germ cell-specific RNAs, RBPs, and translational regulators. Germ granules are involved in germ cell development and germline specification. They play roles in RNA regulation, mRNA localization, and translational control within germ cells.^151^

### Nuclear speckles

Nuclear speckles are found in the nucleus. They are abundant in pre-mRNA splicing factors, RNA polymerase II, and transcriptional regulators. Nuclear speckles are evolving complexes involved in the storage and assembly of pre-mRNA splicing factors.^152^ They also regulate gene expression and mRNA processing.^153^

These are just a few examples of MLOs found in cells. Each organelle has unique compositions and functions, contributing to different aspects of cellular physiology, including gene expression regulation, RNA metabolism, and stress responses.

## MLOs and biomolecule condensates in physiological conditions

**Fig. 4:**
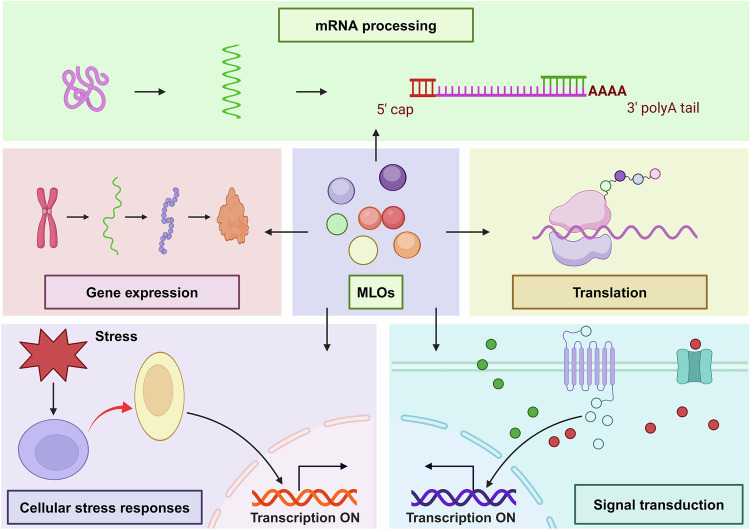
Biological functions of MLOs. The biological functions of MLOs include gene expression, mRNA processing, translation, cellular stress responses, and signal transduction

MLOs have garnered significant attention in cell and molecular biology owing to their roles across different normal physiological states, such as gene expression, mRNA processing, translation, stress response and signal transduction (Fig. 4). For example, the nucleolus orchestrates the intricate process of ribosome assembly. The nucleolus and P-body are involved in the regulation of stem cell fate decision. Additionally, following cellular stress or viral infections, cells form SGs, which are assemblies of RNA-binding proteins, ribosome subunits, and stalled mRNAs following the general arrest of protein translation.

### Stem cell fate determination and embryonic development

Stem cells have the potential to differentiate into different types of cells. Recent studies suggest that LLPS participates in asymmetric cell division,^154^ where two daughter cells with distinct fates are produced. Stem cells use asymmetric division for self-renew and generate specialized daughter cells. The capacity of stem cells to undergo asymmetric division is essential for the diversity of cell types and tissue maintenance. LLPS facilitates the polarized distribution of proteins during the asymmetric division of *Drosophila* neuroblasts.^155–157^ In *Drosophila* neuroblasts, the Par complex undergoes condensation dependent on the cell cycle, facilitated by LLPS. Disruptions in the phase separation of Par3/Par6 hinder the formation of apical-basal polarity during asymmetric divisions of neuroblasts, resulting in faulty lineage development.^155^

Recent discoveries propose that nucleoli actively regulate pluripotency and differentiation by influencing the expression of key regulatory genes.^158^ Stem cell self-renewal entails the division of stem cells to generate additional stem cells, sustaining the stem cell reservoir over time. Although the nucleolus is implicated in this crucial process, the precise molecular mechanisms governing it remain elusive. TMF1-regulated nuclear protein 1 (Trnp1) is a low-complexity protein with the ability to regulate the self-renewal of neural stem cells through phase separation.^159^ It was shown that Trnp1 maintains neural stem cells in a self-renewal proliferation state by interacting with factors present in various nuclear MLOs, including the nuclear speckles, nucleolus, and condensed chromatin.^160^ Reducing Trnp1 levels in mice have been demonstrated to decrease glia cell proliferation while concurrently enhancing their differentiation.^159^ This is the first nuclear protein that regulates stem cell fate by organizing the size, structure, and function of various MLOs.

Nucleolin, the principal nucleolar protein in actively dividing eukaryotic cells, is primarily known for its role in ribosome biogenesis. The precise localization of nucleolin proves to be crucial for myogenic differentiation. An anti-nucleolin aptamer, iSN04, was able to induce the differentiation of induced pluripotent stem cells (iPSCs) into Nkx2.5^+^ beating cardiomyocytes.^160^ iSN04 forms a guanine quadruplex-like structure, which was recognized by the RNA-binding domains of nucleolin. This unique conformation of iSN04 is pivotal for facilitating cardiomyogenesis.^160^ These findings are significant as iPSCs derived from patients offer immune-compatible cell sources for transplantation. Nucleolin can also be induced by β-crystallin B2 (CRYβB2) to promote the proliferation of cancer stem cells. In breast cancer cells, the increased expression of CRYβB2 correlates with enhanced stemness, growth, and metastasis.^161^ Within tumors, CRYβB2 fosters de-differentiation, amplifies mesenchymal markers, and promotes the presence of cancer-associated fibroblasts, along with an enlargement of nucleoli. CRYβB2 initiates nucleolin expression, subsequently activating AKT and EGFR signaling pathways.^161^

The nucleolus is also involved in embryonic development. Embryonic cells at the two-cell (2C) stage have totipotent potential with the capability to differentiate into the full range of cell types. Within the mouse embryonic stem (mES) cell cultures, there is a subset of cells that can spontaneously transition into a 2C stage embryo.^162^ However, the precise molecular mechanisms driving this transition remain elusive. Recent research has demonstrated that CX-5461, an agent that can induce nucleolar stress by inhibiting RNA polymerase I (Pol I), can promote the expansion of 2C-like cell population.^163^ A recent study confirmed the significance of nucleolar phase separation in determining stem cell fate.^132^ It demonstrated that the nucleolus-localized RBP LIN28A undergoes LLPS in both mES cells and in vitro conditions, and the ability of pluripotent cells to transition between states relies on this capacity for phase separation.^164^

The fate of cells is regulated by the modification known as N6-methyladenosine (m6A). It was shown that the LLPS phenomenon involving YTH N6-methyladenosine RNA-binding protein 1 (YTHDF1), a crucial “reader” protein for m6A, plays a significant role in driving spermatogonial stem cells to undergo transdifferentiation into cells resembling neural stem cells.^165^ This process is facilitated by activating the IκB-nuclear factor κB (NF-κB)-CCND1 pathway.^165^ Cell fate is also influenced by topologically associating domain (TAD), which is a region of the genome that interacts with itself. Changes in TAD organization might influence cell fate changes by controlling important genes that determine cell identity.^166^ However, the precise relationship between the reorganization of TAD and cell fate decision remains unclear. Recent research has discovered that TADs undergo reorganization during cellular reprogramming, which is linked to phase separation of the pluripotent protein OCT4.^167^

P-bodies contribute to stem cell decision-making by controlling the stability and translation of mRNAs, mediating miRNA activity, responding to cellular stress, interacting with key signaling pathways, and potentially influencing epigenetic regulation.^168^ Kedia et al. demonstrate that, during the development of murine cerebral cortex, the assembly of P-body promotes neural stem cell self-renewal. They further showed that the ubiquitination of 4E-T leads to the assembly of P-body in neural progenitor cells.^169^ Notably, 4E-T inhibits translation to ensure the stem cell pool is not depleted during a period of rapid cell genesis.^170^ However, in mES cells, increased levels of P-body primes mES cells for differentiation.^171^ Mechanistically, the O-GlcNAc modification of proteasome activator subunit 3 (Psme3) enhances the breakdown of DEAD box polypeptide 6 (Ddx6), an essential component for P-body assembly, thereby maintaining mES cells pluripotency. Conversely, Ddx6 is stabilized in the absence of Psme3 O-GlcNAcylation, leading to the spontaneous transition of mES cells out of the pluripotent state. These findings indicate that P-bodies play different roles in cell fate decisions depending on cell types.^171^

### Regulation of gene expression and RNA metabolism

MLOs play a critical role in regulating gene expression and RNA metabolism at various levels, including transcription, RNA processing, and translation. Studying these dynamic structures provides valuable insights into their functions in health and disease, potentially leading to new therapeutic interventions.

#### Transcription regulation

The nucleolus is essential for rRNA synthesis and ribosome assembly, influencing rRNA gene transcription by concentrating the necessary machinery and substrates. The transcriptional activity within the nucleolus is linked to its internal pH, which regulates the recruitment and condensation of the DEAD-box RNA helicase DDX21.^111^ Nuclear speckles, which contain pre-mRNA splicing factors, play an important role in mRNA processing. The dynamic three-dimensional spatial organization of genomic DNA drives the high concentrations of splicing factors within these nuclear speckles.^172^

#### RNA processing and modification

CBs participate in regulating the maturation and assembly of RNPs, which are crucial for splicing pre-mRNA. These bodies form at specific locations within the genome due to high transcriptional activity. Depletion of CBs disrupts splicing dynamics by inhibiting the transcription of small nuclear RNA (snRNA) and small nuclear RNPs (snRNPs).^173^ P-bodies play a significant role in mRNA decapping and degradation, thereby affecting mRNA turnover and gene expression levels. These dynamic cytoplasmic MLOs contain components for mRNA storage and degradation, such as deadenylase and decapping factors. Additionally, various mRNA metabolic regulators, including m6A readers and those involved in miRNA-mediated gene silencing, are linked to P-bodies.^174^

### Signal transduction

MLOs are essential for the spatial and temporal regulation of signal transduction pathways. Their ability to compartmentalize signaling molecules, dynamically assembles and disassembles, and enhances reaction specificity and efficiency makes them integral to cellular signaling. Understanding their roles in health and disease can open new avenues for therapeutic interventions targeting dysregulated signaling pathways.

By concentrating specific signaling molecules, MLOs create microenvironments where signaling reactions can occur more efficiently and with higher specificity. For example, PML-NBs sequester tumor suppressor proteins like p53, regulating their stability and activity. They also manage reactive oxygen species (ROS) homeostasis by linking ROS to p53 signaling, enforcing basal ROS protection, and mediating their acute toxicity.^175^ PML-NBs are also implicated in regulating interferon signaling pathways. Upon infection with IE1-deficient HCMV, PML-NBs rearrange into enlarged PML cages. This process requires interferon signaling and DNA damage response, causing the invading HCMV genomes to become trapped within PML-NBs in a transcriptionally repressed state. This functions as a defensive approach to combat viral infections by combining interferon and DNA damage signaling to capture both nucleic acids and protein components.^126^

SGs modulate signaling pathways implicated in the cellular stress reaction by sequestering key signaling molecules and mRNAs, thereby regulating their translation and activity. The stress reaction induced by nucleic acids is vital for antiviral defense and innate immunity. SARS-CoV-2 evades the immune response by attenuating antiviral SG formation.^176^ SG assembly inhibits apoptosis and enhances cell survival under stress. Using a proximity-labeling technique, Fujikawa et al. demonstrated that the buildup of caspase-3/7 in SGs is necessary to inhibit caspase activation and prevent apoptosis.^177^

## MLOs and biomolecule condensates in diseases

Dysfunctional MLOs and condensates have the capacity to interfere with protein localization, signal transduction, and gene expression, ultimately contributing to the development of various diseases.

### Cardiovascular diseases

The condensates formed *via* LLPS are vital in organizing signaling molecules and transcription factors that participate in cardiac differentiation and remodeling. An imbalance in phase separation has been linked to cardiomyopathies and heart failure.^178,179^ Grasping the fundamentals of LLPS within cardiac biology presents a transformative perspective for understanding the molecular complexities underlying heart health and disease.

**Fig. 5:**
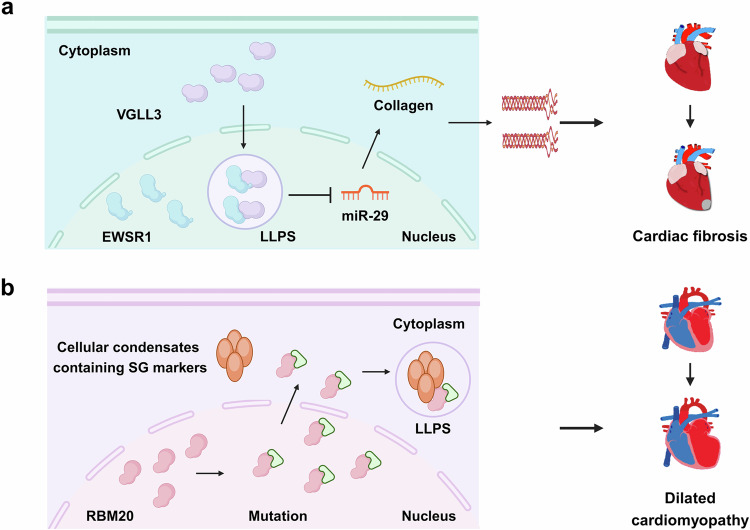
The role of MLOs in cardiovascular diseases. **a** Within the nucleus, VGLL3 undergoes LLPS facilitated by its LCD, forming condensates alongside the EWSR1, which inhibits miR-29, leading to increased collagen production and cardiac fibrosis. **b** The mutated RBM20 from nuclear splicing speckles relocates to SG, leading to dilated cardiomyopathy

Myofibroblasts play a pivotal role in causing cardiac fibrosis by producing collagens. Collagen production can be induced by TGF-β and matrix stiffness. Vestigial-like family member 3 (VGLL3) is a protein sensitive to mechanical stimulation^180^ implicated in myogenesis,^181^ cell proliferation^182^ and autoimmune disorders.^183^ Recent studies have revealed that substrate stiffness can cause VGLL3 to translocate to the nucleus to induce the production of collagen.^184^ Within the nucleus, VGLL3 undergoes LLPS facilitated by its LCD (Table 2), forming condensates alongside the non-paraspeckle NONO condensates possessing the EWS RNA-binding protein 1 (EWSR1).^184^ Upon binding to EWSR1, VGLL3 effectively suppressing miR-29b, a molecule that inhibits collagen production.^184^ Consistent with these findings, cardiac fibrosis is notably diminished in VGLL3-knockout mice, accompanied by increased expression of miR-29b after infarction (Table 2).^184^ These reports suggest that VGLL3 phase separation is implicated in the development of cardiac remodeling and heart failure (Fig. 5).

**Table 2:** Proteins form LLPS in the cardiovascular system

Name	Disease	Mechanism	Reference
HIP55	Heart failure	Phase separation of HIP55 relies on Akt-mediated phosphorylation; prolonged sympathetic stimulation and stress inhibit the phosphorylation, leading to dysregulated phase separation and aggregate formation.	^179^
RBM20	Dilated cardiomyopathy	The pathogenic R636S variant of RBM20 induces abnormal accumulation of RNP granules in the sarcoplasm.	^187^
VGLL3	Cardiac fibrosis	VGLL3 (LCD, aa63-78) is translocated to the nucleus triggered by substrate stiffness and undergoes LLPS, which promotes collagen production by suppressing miR29b.	^184^

RNA-binding motif protein-20 (RBM20) is a splicing factor highly expressed in the heart.^185^ Linkage analysis has revealed that RBM20 mutations are linked to dilated cardiomyopathy (DCM), highlighting a mutation hotspot in the arginine/serine (RS)-rich region.^186^ This mutation is characterized by a defect in RNP condensates, leading to abnormal heart development and function.^154^ The mutations cause RBM20 to relocate from the nucleus to cytoplasm and merged with other components within the SGs.^187^ This condensatopathy results in the restriction and isolation of polysomes, mRNA, and cytoskeleton proteins. Therefore, DCM caused by RBM20 has been considered a RNP granule disease (Fig. 5),^187^ which may be cured by either antisense oligonucleotides or adeno-associated virus-mediated gene therapy.^185^

### Neurodegenerative diseases

**Fig. 6:**
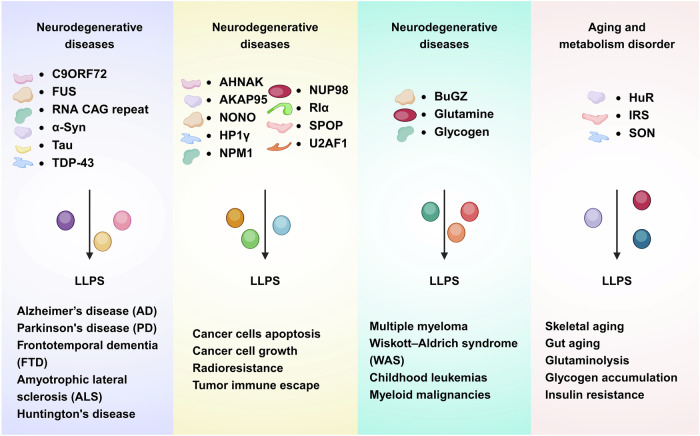
Diseases linked to dysregulation of MLOs. Abnormal phase separation or dysfunction of specific biomolecular condensates can disrupt cellular homeostasis and contribute to a range of diseases, including neurodegenerative diseases, cancer, hematological diseases, aging and metabolism disorder

Neurodegenerative diseases present a significant global health challenge, imposing a growing burden on individuals and societies. Recent progress in cell biology has underscored the importance of MLOs in neurodegeneration. These dynamic structures, shaped through LLPS, have become pivotal components in the complex network of cellular dysfunction linked to disorders such as Amyotrophic lateral sclerosis (ALS), Frontotemporal dementia (FTD), Parkinson’s disease (PD), Alzheimer’s disease (AD), and Huntington’s disease (Fig. 6).^188–190^

Tau, a crucial neuronal protein implicated in AD, typically resides in axons under normal physiological conditions, participating in microtubule assembly.^191–195^ In tauopathies, it becomes hyperphosphorylated and detaches from the microtubule, exhibiting amyloid fibril characteristics.^191–194,196,197^ Recent studies highlight that purified full-length tau (tau441) readily undergoes LLPS in vitro, especially when crowding agents are present to mimic cellular macromolecule concentrations (Table 3). This LLPS phenomenon occurs regardless of tau phosphorylation status, driven mainly by interactions between N-terminal and C-terminal regions of tau through electrostatic attraction.^198–202^ Additionally, tau condensation into droplets is facilitated by polyanions such as heparin or RNA.^203,204^ These in vitro observations find partial support in cellular studies.^205–209^ Contrary to previous suggestions, a recent study showed that point mutations in the pseudorepeat region do not significantly alter tau’s tendency for LLPS.^210^ However, these mutations notably accelerate liquid-to solid phase transition and promotes the formation of fibrillar aggregate.^210^ These studies suggest that various forms of interactions are involved in phase separation and subsequent fibrillar aggregate formation.^211^ Studies using electron cryo-microscopy showed that tau filaments form distinct structures in different neurodegenerative diseases.^212^ Further studies are needed to find out whether different tau isoforms or mutants are involved in forming these distinct structures and whether other co-factors are involved in assembling the filaments.

**Table 3:** Proteins form LLPS in neurodegenerative diseases

Name	Disease	Mechanism	Reference
C9ORF72	ALS/FTD	Expansion of GGGGCC repeats in the gene C9ORF72 alters its LLPS dynamics	^247^
FUS	ALS	Wild-type FUS forms reversible fibrils	^256^
		FUS with glycine mutations undergo rapid loss of fluidity	^240^
RNA CAG repeat	Huntington’s disease	RNA molecules with expanded CAG repeat (eCAGr) form cytoplasmic gel-like foci that significantly reduce the global protein synthesis rate	^248^
αS (α-synuclein)	Parkinson	Phase-separated droplet forms a precursor for the pathogenic αS fibrils.	^190^
Tau	Alzheimer	The phosphorylated or mutant tau can initiate aggregation through LLPS	^198^
		The microtubule-binding repeats of Tau form liquid droplets in a phosphorylation-specific manner.	^197^
TDP-43	ALS	Two familial variants within the^312^ NFGAFS^317^ segment of TDP-43(A315T and A315E), together with phosphorylation, create pathogenic aggregation.	^224^

Alpha-synuclein (α-syn) is a synuclein protein mainly found in neurons and is involved in the pathogenesis of PD, which manifests as a multisystem disorder with a spectrum of motor and non-motor symptoms. Pathologically, these manifestations are linked to extensive aggregated proteins known as Lewy bodies (LBs) within neurons.^213^ A key constituent of LBs is α-syn encoded by the SNCA gene.^214^ Amplification of the wild-type SNCA gene leads to early-onset PD and dementia.^215^ Various investigations have suggested that aggregates formed by misfolded α-Syn are implicated in cell death during PD progression.^216^ It was shown that α-Syn has the ability to undergo LLPS, typically resulting in the formation of amyloid fibrils (Table 3).^217^ Recent findings indicate that LLPS of α-Syn takes place during the nucleation phase of aggregation. The α-Syn droplets with liquid-like properties undergo an irreversible shift into amyloid-like hydrogels, encapsulating oligomers and fibrils.^218^ Research also demonstrated that α-Syn directly influences P-bodies, which are MLOs responsible for mRNA turnover and storage. The α-Syn binds with various decapping proteins closely positioned on the Edc4 scaffold. Elevated levels of α-Syn, as observed in pathological conditions, lead to increased association with Edc4, thereby interfering with interactions with other decapping-module proteins. Consequently, certain mRNAs, such as those involved in protein trafficking and RNA metabolism, are stabilized.^219^ Questions remain as to why only a selected group of mRNAs are stabilized and how they contribute to the formation of LBs. Although highly expressed in neurons, α-Syn is also found in other tissues such as blood^220^ and kidney.^221^ So the question is why only neuron is affected.

Neurons efficiently transport all essential components for translation, such as ribosomes, mRNA, and translation factors, to synthesize proteins at distant locations. The localization pattern of mRNAs is tightly regulated by several RBPs, such as FUS, TDP-43, and hnRNPA1, which are associated with ALS. TDP-43 and FUS can form condensates with various material conditions involved in both normal cellular process and disease. These RBPs play pivotal roles in regulating pre-mRNA splicing and transcription. Moreover, they constitute constituents of RNP granules triggered by stress, comprising RBPs and RNA, and are prominently present in cytoplasmic aggregates within neurons in degeneration, serving as essential disease markers of ALS and FTD.^222^

The presence of pathological aggregation of phosphorylated TDP-43 (p-TDP-43) is a key feature of ALS and FTD. ALS stands as the predominant clinical manifestation of upper and lower motor neuron disease.^223,224^ A mounting body of evidence substantiates the concept of overlapping genetic and pathological features between ALS and FTD.^225^ The pathology of both ALS and FTD has been associated with environmental factors and an array of genetic changes, encompassing multiple point mutations in the LCR of proteins localized within RNP granules, as well as repeat expansions.

TDP-43 is involved in many cellular activities, such as regulating mRNA splicing, RNA transportation, and forming cytoplasmic SGs that halt translation.^226^ These functions often occur within RNP granules, which are formed through LLPS.^227^ In typical physiological circumstances, TDP-43 is mainly found in the nucleus, forming oligomers and residing within biomolecular condensates assembled through LLPS. However, in disease states, TDP-43 forms inclusions either in the cytoplasm or intranuclearly. Studies indicate that the carboxy-terminal domain of TDP-43 alone can induce phase separation. Mutations linked to ALS interfere with interactions and hinder this phase separation process, potentially explaining the functional impairment observed.^228^ The significance of TDP-43 in pathological condition is emphasized by the fact that dominant missense mutations alone are adequate to induce disease. Studies have revealed that cytoplasmic RNP granules formed by TDP-43 facilitate the delivery of target mRNA to distant neuronal regions through microtubule-dependent transport system. The ability to transport mRNA is impaired by TDP-43 mutations that cause ALS. Thus, TDP43 mutations associated with ALS result in a partial loss of the physiological function of TDP-43 (Table 3).^229^

By conducting whole genome linkage analysis and exome sequencing, Ervilha Pereira et al. identified a frameshift mutation in TDP-43 that was conclusively linked, resulting in a C-terminally altered PrLD (TDP-43p.Trp385IlefsTer10). Muscle biopsies obtained from patients showcased TDP-43-positive sarcoplasmic inclusions. In vitro phase separation assays revealed that TDP-43Trp385IlefsTer10 formed solid-like fibrils instead of liquid-like condensates, indicating an increased tendency for aggregation compared to wild-type TDP-43. Collectively, these findings affirm that TDP-43p.Trp385IlefsTer10 is a partial loss-of-function and susceptible to aggregation variant responsible for autosomal dominant vacuolar myopathy.^230^

A lingering question has revolved around the consequences of the processes involved in TDP-43 mutations and how their impacts correspond to the events accompanying the pathological re-localization of TDP-43 in patients. TDP-43 WT RNP granules display discrete biophysical characteristics according to where they are situated in the axon, while granules generated by ALS-associated mutant TDP-43 exhibit increased viscosity and impaired axonal transport function.^231^ Through the utilization of various cellular systems expressing variants of TDP-43 based on structure, Perez-Berlanga et al. demonstrated that oligomerization and RNA binding play pivotal roles in governing the stability of TDP-43, its splicing activity, LLPS, and cellular distribution.^232^ As an RNA-binding protein, mislocalization of TDP-43 could potentially alter RNA metabolism. However, TDP-43-regulated RNAs in motor neurons are yet to be discovered. In this context, Klim et al. illustrated that the expression of STMN2, a microtubule-modulating protein crucial for normal axonal expansion and regrowth, decreased following TDP-43 silencing, aberrant TDP-43 distribution, and spinal cords from deceased patients.^233^ Neuropathological investigations substantiate the notion that TDP-43 aggregates might spread from one cell to another, leading to the dissemination of pathological inclusions in the brain. The transmission of TDP43 from cell to cell has been observed in cell cultures, potentially contributing to the pathological propagation of TDP43 in FTLD-TDP.^234^ A recent study confirmed these findings in vivo, demonstrating that a single intracerebral injection of pathological TDP43 derived from human brains affected by FTLD-TDP initiates the onset and propagation of TDP43 inclusions in the brain in a spatiotemporal-dependent manner.^235^

Using the optogenetic platform, Mann et al. showed that the generation of pathologically relevant TDP-43 phase transitions can be prevented by adding a “bait” RNA oligonucleotide.^236^ Compared to the antisense oligonucleotides, which suppress protein expression, the “bait RNA” regulates protein solubility via reversible interaction; therefore, it is a useful approach for treating either gain- or loss-of-function protein aggregates.

Although it is generally believed that the generation of insoluble protein aggregates is responsible for the development of neurodegenerative diseases, a recent study showed that some mutations that increase TDP-43 aggregation actually decrease toxicity in yeast cells by titrating proteins away from toxic interactions.^237^ Therefore, this yeast cell toxicity model may be useful to evaluate antisense or “bait RNAs” before initiating clinical trials.

Uncommon mutations affecting the LCD of the RBP T-cell-restricted intracellular antigen-1 (TIA1) have been detected in individuals with ALS and FTD. TIA1 holds an important position as an SG constituent, and its LCD is essential for SG congregation. The mutations associated with the disease affect the biophysical behavior of TIA1, enhancing its tendency for phase separation, causing a delay in SG disassembly, and fostering the buildup of static SGs. These SGs house TDP-43, which experiences reduced mobility and increased insolubility.^238^ Therefore, it was suggested that delayed disassembly of SG might increase the accumulation of insoluble TDP-43.^238^ Along this line, it was shown that TIA1 facilitates the phase separation and formation of toxic tau.^239^

Fused in sarcoma (FUS), a nuclear RBP, manifests its pathogenic signature in ALS and FTD through cytoplasmic aggregation. FUS is involved in various RNA metabolism and the assembly of RNP bodies, including SGs. These RNP bodies are thought to arise through LLPS, driven by temporary RNA and RBP interactions that possess IDRs and RNA recognition motifs (RRMs). FUS forms fluidic structures at DNA breakpoints in cells and in the cytoplasm during stress, and FUS liquid droplets transition over time from a fluid to an aggregated state, a process accelerated by mutations observed in patients (Table 3).^34^ The mechanisms by which FUS-RNA interactions drive phase separation and whether ALS-associated mutations affect this behavior are not fully understood. A recent study showed that wild-type FUS binds single-stranded RNA in a manner dependent on length, forming small, fluid condensates through dynamic interactions with RNA multimers.^240^ In contrast, glycine mutations in FUS result in a rapid decrease in fluidity, underscoring the pivotal role of glycine in promoting fluidity.^240^ Interestingly, although both FUS and TDP-43 can form aggregates in SGs, they do so in a mutually exclusive manner.^241,242^ The mechanisms responsible for this phenomenon remain incompletely understood but may be due to different “molecular grammars” involved in their phase separation.

Another RBP that is recruited to SGs is hnRNPA1,^243^ a part of the heterogeneous nuclear ribonucleoproteins (hnRNPs) family, which predominantly functions as nuclear RBPs, forming complexes with RNA polymerase II transcripts. These proteins engage in diverse cellular activities, including transcription, pre-mRNA processing, and translation. Recent investigations propose that numerous intrinsic features of hnRNPs contribute to their participation in various regulatory pathways.^244^ Genetic evidence establishes a connection between persistent SG and the buildup of abnormal inclusions.^33^ The disease-associated hnRNPA1 undergoes LLPS, forming protein-rich droplets mediated by LCD.^33^ When SGs consist of RBPs containing LCD mutations that promote fibrillization or when SGs persist as a consequence of interruptions in the disassembly process, pathogenic fibrils can form and evade the surveillance of quality control.^43^ Similar to disease-causing mutations observed in hnRNPA1, the variants D290V and P298L promote aggregation in hnRNPA2.^245^ Interestingly, deficiency of nuclear hnRNPA1 in motor neurons alongside concurrent cytoplasmic aggregation of TDP-43 is a key feature in progressive neuronal death in ALS.^246^ These findings suggest that the disappearance of nuclear hnRNPA1 might be related to TDP-43 in the SGs.

The occurrence of repeat expansions of small nucleotide segment represents another unique type of genetic modification linked to diseases like Huntington’s disease, myotonic dystrophy, ALS/FTD, and spinocerebellar ataxias. The extent of these illnesses is proportional to the repeat’s length. The resultant abnormal polypeptides or RNAs form condensates and recruit other crucial molecules. One example is the GGGGCC repeat, a common cause of ALS/FTD. The buildup of the repeat that contains RNAs within nuclear regions might drive the disease by sequestering RBPs.^247^ Another example is the expanded CAG repeats (eCAGr). RNA molecules carrying eCAGr have the potential to undergo sol-gel phase transitions and form cytoplasmic gel-like foci, which may substantially decrease the rate of protein production, possibly through the sequestration of the elongation factor eEF2 for translation. In brain tissue sections from a knock-in mouse model and from patients affected by Huntington’s disease, eEF2 puncta were notably enhanced. Furthermore, the injection of adeno-associated virus containing eCAGr RNA resulted in significant behavioral impairment in mice.^248^

In summary, there is substantial evidence indicating a strong association between the LLPS of specific proteins and the progression of neurodegenerative diseases.^211,249–255^ However, the precise molecular basis for this connection remains inadequately understood. A significant argument supporting a clear mechanistic link between LLPS and the development of disease is the observation that mutations in proteins such as TDP43,^228^ FUS,^34,256^ hnRNPA1,^33^ hnRNPA2,^245^ or TIA1^238^ induce abnormality of MLOs within cells or liquid droplets formed in laboratory settings. Nevertheless, the nature of these abnormalities appears to vary depending on the specific protein and mutation involved. For instance, some mutations, like those in TIA1, promote LLPS,^238^ while others, notably most mutations in TDP43, have the opposite effect.^228^ Moreover, the implications of LLPS in terms of protein aggregation also differ depending on the protein in question.^253^

### Cancer

Recent advancements have unveiled a connection between abnormal phase separation and various types of cancer. The abnormal phase separation leads to genomic instability and disruption of transcription and signal transduction.^257–259^

p53 is a tumor suppressor protein involved in many signaling pathways in cells under stress. The p53-binding protein 1 (53BP1) is an interactor of p53.^260^ Ghodke et al. identified AHNAK as a scaffolding protein that binds to 53BP1. AHNAK prevents overactive interaction between 53BP1 and p53. This regulatory mechanism protects cancer cells from apoptotic stimuli. The loss of AHNAK results in enhanced p53-mediated apoptosis due to excessive buildup of 53BP1 on chromatin and enhanced phase separation (Table 4).^260^ Thus, AHNAK functions as a rheostat of p53 by restraining 53BP1 phase separation.

**Table 4:** Proteins form LLPS in cancer

Name	Disease	Mechanism	Reference
AHNAK	Cancer cell survival	AHNAK is a G1-enriched interactor of 53BP1 that ensures optimal partitioning of 53BP1 into phase-separated condensates and limits excessive interaction with p53, which leads to apoptosis in cancer cells	^260^
AKAP95	Cancer	AKAP95 is a nuclear protein that regulates transcription and RNA splicing by forming liquid-like condensates in nucleus.	^261^
HP1γ	Myeloma	The deacetylation of HP1γ promotes nuclear condensation, and this condensed form of HP1γ plays a crucial role in drug resistance by facilitating DNA repair in multiple myeloma cell.	^286^
NONO	Tumor radioresistance	LLPS of NONO recruits nuclear EGFR and DNA-PK and promotes DNA repair, leading to radioresistance.	^262^
NPM1 (Nucleophosmin)	Triple-negative breast cancer (TNBC)	NPM1 undergoes LLPS through interactions with nucleolar components, including rRNA and proteins featuring multivalent arginine-rich linear motifs (R-motifs). NPM1 binds to the PD-L1 promoter in TNBC cells, activating PD-L1 transcription.	^263,264^
NUP98 (Nucleoporin 98)	Leukemia	The biomolecular condensation is embedded within the N-terminus of NUP98 and possesses the ability to induce leukemia-specific gene expression.	^290^
RIα (Type I regulatory subunit of PKA)	Cell transformation	Loss of RIα LLPS in normal cells induces cell transformation.	^44^
SPOP	Prostate, breast cancer	Cancer-associated mutations in tumor suppressor SPOP disrupt LLPS and correlate with a loss of function.	^275^
U2AF1	Myeloid malignancies	U2AF1 splicing factor mutations, lead to an increased SG response, indicating a new function for biomolecular condensates in adaptive oncogenic mechanisms.	^292^

AKAP95, a nuclear protein, is overexpressed in clinical samples of cancer tissues. AKAP95 contributes to tumor growth by facilitating the splicing of cyclin A2, an important regulator of the cell cycle.^261^ The regulatory functions of AKAP95 in gene expression and tumorigenesis are contingent on its capacity to establish condensates with appropriate liquidity and dynamicity (Table 4).^261^ The data suggest that AKAP95 is involved in tumorigenesis by promoting the proliferation of cancer cells. Radioresistance stands out as a primary contributor to the failure of cancer treatment, resulting in relapse and diminished survival outcomes for cancer patients. NONO, an RNA/DNA-binding protein with LLPS capability, has become an essential regulator of tumor radioresistance. The LLPS of NONO facilitates the recruitment of nuclear EGFR and DNA-PK, intensifying their interaction. This cascade leads to an augmented initiation of DNA damage-induced pT2609-DNA-PK and fosters non-homologous end joining (NHEJ)-mediated DNA repair, ultimately culminating in tumor radioresistance. The phase separation-mediated radioresistance mediated by NONO presents a potential novel molecular target for sensitizing tumor cells to radiotherapy (Table 4).^262^

The interaction between programmed cell death protein-1 (PD-1) and its ligand (PD-L1) is pivotal in tumor immune escape mechanisms. Triple-negative breast cancer (TNBC) exhibits elevated expression of PD-L1 compared to other subtypes. Nucleophosmin (NPM1) activates the transcription of PD-L1 in TNBC cells by specifically binds to its promoter. Consequently, this activation hinders T-cell activity both in vitro and in vivo.^263^ NPM1, a highly prevalent oligomeric protein located in the nucleolus, is actively involved in ribosome biogenesis by interacting with nucleolar components through self-interaction mediated phase separation (Table 4).^264^

Oncogenic receptor tyrosine kinase fusion proteins exhibit elevated assembly, forming membraneless cytoplasmic protein granules. These granules play a crucial role in coordinating local RAS activation and organizing RAS/MAPK signaling within lung cancer cells.^70^ Additionally, RIα, the type I subunit of cAMP-dependent protein kinase A (PKA), undergoes LLPS in response to cAMP signaling, leading to the generation of cAMP-enriched biomolecular condensates (Table 4). Normal cells use this LLPS process to sequester and constrain cAMP. However, in the presence of PKA fusion oncoprotein, the phase separation of Riα is blocked, leading to enhanced cell proliferation and the induction of cell transformation.^44^

RNA-binding protein 14 (RBM14) is a coactivator of nuclear receptors, and its expression is increased in castration-resistant prostate cancer (CRPC). Despite androgen deficiency, the androgen receptor signaling pathway remains an important driving force in CRPC. Tsuji et al. demonstrated that RBM14 promotes phase separation, sustaining prostate-specific antigen expression during androgen suppression in human prostate cancer.^265^

SPOP (speckle-type POZ protein) is a cancer inhibitor, and its mutations cause solid tumors.^266–270^ SPOP functions as a binding scaffold of the cullin3-RING ubiquitin ligase and attracts substrate through LLPS.^271–274^ Cancer-associated mutations in SPOP impair LLPS and are associated with a loss of function (Table 4).^275^

UTX/KDM6A, a gene encoding a histone H3K27 demethylase, acts as a crucial cancer suppressor commonly mutated in human cancers.^276^ However, subsequent studies revealed that the demethylase activity of UTX is frequently independent of its role as a tumor suppressor.^277–282^ A recent study revealed that the chromatin regulatory activity of UTX in tumor suppression is rooted in its phase separation.^283^ The core intrinsically disordered region (cIDR) of UTX gives rise to condensates through phase separation, the loss of cIDR due to mutations is a key factor in nullifying tumor suppression.^283^

### Hematological disorders

In hematological disorders, which encompass a diverse range of conditions affecting blood cells and their precursors, recent progress in cell biology has brought attention to the crucial role of MLOs in orchestrating cellular processes. This provides novel insights into the pathophysiology of blood diseases.

Heterochromatin protein 1γ (HP1γ) is a reader of H3K9me2/3^284^ that is involved in efficient transcriptional elongation.^285^ A significant challenge in managing multiple myeloma is the resistance to proteasome inhibitors. Elevated HP1γ levels are linked to poorer clinical outcomes.^286^ Mechanistically, heightened HDAC1 activity in bortezomib-resistant myeloma cells leads to the deacetylation of HP1γ at lysine 5, promoting nuclear condensation. This condensed form of HP1γ is essential for chemotherapy resistance in multiple myeloma cells (Table 4). These findings suggest that targeting HP1γ could effectively overcome drug resistance in patients with relapsed multiple myeloma.^286^

Wiskott–Aldrich syndrome protein (WASP) is an actin nucleation factor.^287^ Mutations in WASP cause Wiskott–Aldrich syndrome (WAS), which is an X chromosome-linked disease characterized by thrombocytopenia, eczema, and immune deficiency.^288^ WASP controls the transcription of splicing factors via phase separation. Mutations in the WASP gene result in abnormal RNA splicing.^289^

The nucleoporin 98 (NUP98) protein is a member of the nuclear pore complexes that regulate the movement of macromolecules between the nucleus and cytoplasm. NUP98 gene is recurrently translocated, particularly in pediatric leukemia cases. Terlecki-Zaniewicz et al. explored the protein interactomes of several NUP98 proteins in human leukemia cells using confocal imaging. They demonstrated that various NUP98 fusion proteins with distinct structures are concentrated within biomolecular condensates by LLPS. Additionally, they showed that the structural characteristics directing the condensation are embedded within the N-terminus of NUP98 and possess the ability to trigger leukemia-specific gene expression when incorporated into oncogenic fusion proteins (Table 4).^290^ These observations were confirmed by a subsequent study showing that NUP98 fusion oncoproteins contain an LCR that is prone to LLPS, driving the transformation of hematopoietic cells.^291^

Mutations in splicing factors, particularly U2AF1, have become prevalent key factors in myeloid malignancies. However, the precise influence of these mutations on splicing and their role in promoting cancer has remained ambiguous. Biancon et al. utilized high-resolution interactome to reveal that U2AF1 splicing factor mutations lead to an intensified stress response in myeloid malignancies.^292^ Cell lines carrying U2AF1 mutations and blasts derived from MDS/AML patients exhibited an increased SG response, indicating a new function for biomolecular condensates in adaptive oncogenic mechanisms (Table 4).

### Aging and metabolism disorder

Some studies suggest that changes in the composition, dynamics, or functionality of MLOs could contribute to aging-related cellular dysfunction by altering the content or the formation of SGs.^293^

Human antigen R (HuR) is a bone-associated RBP that forms SGs. Research has demonstrated that both the expression of HuR and the generation of HuR-positive SGs decrease in primary osteoblasts obtained from aging mice.^294^ Inhibiting the HuR-positive SGs led to a reduction in osteoblastic differentiation, indicating the vital involvement of HuR and SGs in osteogenesis (Table 5).^294^ These findings underscore the importance of HuR and the associated SGs in enhancing bone formation in skeletal aging and provide a basis for developing innovative therapeutic approaches for age-related skeletal diseases.

**Table 5:** Proteins form LLPS in metabolism and aging

Name	Disease	Mechanism	Reference
BuGZ (a coacervating mitotic effector)	Aging	Inhibiting the phase transition of BuGZ extends the lifespan of *Drosophila*	^295^
Glutamine	Glutamine deprivation stress	When glutamine level is decreased, lncRNA GIRGL inhibits glutaminase activity through phase separation to enable cancer cell survival	^297^
Glycogen	Tumor initiation	Cancer-initiating cells proliferate by increasing glycogen storage and block Hippo signaling through glycogen phase separation.	^298^
HuR (Human antigen R)	Age-related bone loss	HuR is a bone-associated RBP that forms SGs and facilitates osteogenesis during aging	^294^
IRS (Insulin receptor substrates)	Type 2 diabetes	LLPS of IRS functions as intracellular signal hubs to transmit insulin signals	^299^
SON (Nuclear speckle scaffolding protein)	Dysregulated proteostasis	The XBP1s-SON axis dictates a nuclear speckle LLPS dynamics that protects cells from proteome stress	^300^

Research has demonstrated that BuGZ, a coacervating mitotic effector, undergoes condensation in *Drosophila* intestinal stem cells (ISCs) nuclei during interphase, particularly in association with aging and injury. This condensation of BuGZ promotes the proliferation of ISCs, impacting gut repair and longevity in *Drosophila* (Table 5).^295^ Inhibiting the phase transition of BuGZ enhances functions of the intestine and extends the duration of life of these flies. These findings suggest that manipulating protein phase transitions could potentially delay or counteract aging associated with ISCs.^295^

The theory of antagonistic pleiotropy in aging suggests that genes beneficial for early-life fitness may have detrimental effects on lifespan, yet molecular evidence supporting this notion remains largely unexplored. Through a study mapping translatome alterations in Caenorhabditis elegans during the restoration phase after starvation, Wu et al. discovered that trl-1 becomes significantly active after refeeding. Deficiency of trl-1 leads to aberrant upregulation of vitellogenin translation, which enhances reproduction but at the expense of lifespan. They demonstrated that trl-1 undergoes LLPS, forming granules that recruit vitellogenin mRNA and suppress the translation.^296^ These data demonstrate that trl-1 influences the balance between reproduction and longevity by enhancing nutrient provision for the next generation.

MLOs also play important roles in various metabolic processes within the cell by organizing and regulating the dynamics of metabolic reactions across space and time, RNA metabolism, and protein synthesis. Glutamine is a vital supplier of carbon and nitrogen for various biological processes. The initial and crucial step in glutaminolysis is the conversion of glutamine to glutamate, facilitated by the enzyme glutaminase-1 (GLS1). When there is a shortage of glutamine, levels of GLS1 decrease, although the exact mechanisms remain unclear. Research has revealed the existence of a long noncoding RNA called glutamine insufficiency regulator of glutaminase lncRNA (GIRGL), which is upregulated during glutamine deficiency. In the absence of glutamine, elevated levels of GIRGL lead to the formation of a complex between dimers of CAPRIN1 and GLS1 mRNA. CAPRIN1 is a cytosolic phosphoprotein expressed ubiquitously and implicated in various stress responses. This complex formation induces the LLPS of CAPRIN1 and triggers the formation of SG. By inhibiting the translation of GLS1 mRNA, cancer cells can adapt and survive prolonged periods of glutamine deprivation stress.^297^

Cancer cells adapt their metabolic processes to enhance survival and facilitate invasion. Research indicates that glycogen accumulation is crucial in promoting oncogenesis and is likely a prevalent metabolic trait among initially transformed liver cells. The gathered glycogen undergoes LLPS, interfering with Hippo signaling and promoting tumor initiation (Table 5).^298^

Insulin is a metabolic hormone that orchestrates various cellular processes to uphold cell functionality and overall metabolic well-being. The emergence of insulin resistance, characterized by compromised insulin function within cells, is a driving factor behind the escalating global incidence of type 2 diabetes (T2D) in recent decades. Given the widespread prevalence of T2D, an urgent need exists for a comprehensive understanding of the mechanisms governing insulin action and resistance. Upon binding with insulin, the insulin receptor (IR)’s tyrosine kinase activity is activated through autophosphorylation on multiple tyrosine residues, subsequently leading to the phosphorylation of insulin receptor substrates (IRS). Research has revealed that IRS condensates formed *via* LLPS may serve as crucial intracellular signaling hubs, facilitating the transmission of insulin signals deep into the cellular milieu (Table 5).^299^ However, the formation of IRS condensates is impaired in insulin-resistant cells.^299^

Significant strides have been taken in comprehending the properties and functions of biomolecular condensates and LLPS. However, it remains uncertain whether LLPS dynamics are governed by “autonomous clocks.” It was shown that the XBP1s-SON axis incorporates a 12-hour rhythm with LLPS in nuclear speckles to regulate proteostasis across space and time.^300^ These findings indicate that by influencing the timing of proteostasis dynamics, nuclear speckle LLPS could serve as a target for conditions linked to disrupted proteostasis.

## Regulation of LLPS phenomenon

### Regulation by post-translational modifications (PTMs)

**Fig. 7:**
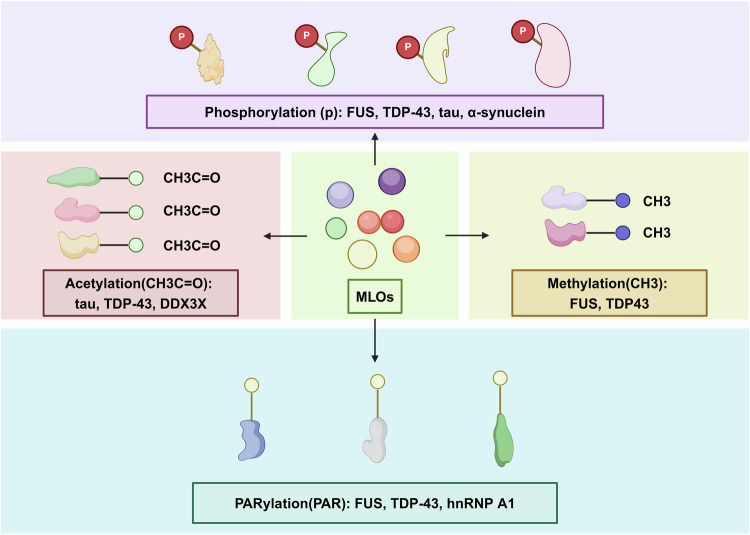
Regulation of MLOs through modifications occurring after translation. The formation and function of MLOs are regulated by modifications occurring after translation, including phosphorylation, acetylation, methylation, and PARylation

PTMs such as phosphorylation,^71^ acetylation,^49^ methylation,^80^ and ubiquitination^301^ can influence gene expression, signaling, and stress responses by modulating the properties of proteins through phase separation, and the recruitment of specific components to the condensates (Fig. 7). PTMs play an essential role in the assembly and disassembly of condensates by introducing covalent changes to protein components.^302^ These modifications significantly influence the ability of IDRs to form condensates.^303^

#### Phosphorylation

Protein phosphorylation involves the attachment of a phosphoryl group, typically to the hydroxyl group of serine, threonine, or tyrosine. At physiological pH, each phosphate group introduces two negative charges to the protein. The heightened negative charge can be harnessed to modify the phase separation of proteins. Notably, serine/threonine phosphorylation of FUS disrupts phase separation and prevents its aggregation.^304^ A phosphomimetic substitution at S48 impairs the polymeric assembly of TDP-43.^305^ These findings reveal the potential for LLPS and aggregation modulation through phosphorylation, presenting intriguing prospects for therapeutics. However, further research is essential to pinpoint the active kinases, delineate the timing and pattern of protein phosphorylation, and elucidate their effects on LLPS-mediated granule formation.

The excessive phosphorylation of the neuronal tau protein stands out as a characteristic feature of Alzheimer’s disease. The phosphorylation of tau by MARK2 was demonstrated to promote K18 LLPS.^197^ An unanswered query revolves around the mechanisms driving tau’s progressive hyperphosphorylation. Recent findings suggest an interdependence mechanism, establishing a connection between initial site-specific and subsequent multi-site phosphorylation. It was shown that a primary phosphorylation site determines the spread of phosphorylation across multiple sites.^306^ It would be interesting to find out whether pathological tau levels can be reduced by inhibiting the master site.

The advancement of Parkinson’s disease hinges significantly on the propagation of pathological α-synuclein (α-Syn). This aggregation of α-Syn propagates in a manner akin to prions, gradually advancing in the brain and from peripheral organs to the brain throughout the disease’s course. Certain proteins on the cell surface, including lymphocyte activation gene 3 (LAG3)^307,308^ and amyloid precursor-like protein 1 (APLP1),^309^ have been identified as receptors facilitating the uptake and spread of α-Syn preformed fibrils (PFFs). However, the specific molecular basis for this preferential binding remains unclear. Recent research suggests that phosphorylation on serine 129 (pS129) enhances the interaction between α-Syn fibrils and these specific receptors.^310^ This discovery is corroborated by the heightened efficiency of pS129 fibrils in cellular uptake, initiation, and induction of PD-like α-Syn pathology in transgenic mice.^310^

##### Acetylation

Lysine acetylation, a reversible PTM, is orchestrated by lysine acetyltransferases and lysine deacetylases, contributing to both histone and non-histone targets. Lysine’s significance in cellular function is underscored by lysine-rich forms of the Alzheimer’s disease-associated protein tau. These variants exhibit coacervation with RNA and are associated with SGs in cellular environments. Acetylation of lysine is demonstrated to reverse LLPS, diminishing the association of tau with SGs.^205^

In the case of TDP-43, acetylation disrupts RNA binding, fostering the buildup of insoluble and hyperphosphorylated TDP-43 forms closely resembling abnormal inclusions in ALS and FTLD-TDP. The presence of acetylated TDP-43 lesions in the spinal cord of ALS patients further suggests a link between abnormal TDP-43 acetylation and the loss of RNA binding in TDP-43-mediated diseases.^309^

Traumatic brain injury (TBI), a prominent risk factor for Alzheimer’s disease (AD), induces tau acetylation at positions similarly acetylated in human AD brain, suggesting a potential molecular connection between TBI and AD.^311^ Targeting post-TBI-induced tau acetylation using acetylation inhibitors, such as salsalate, correlates with diminished neurodegeneration in humans. This approach prevents tau abnormal localization, loss of solubility, and cognitive deficits in animal models, offering potential therapeutic avenues.^311^

Precise control over IDR action is paramount to ensuring that LLPS occurs only when needed. Recent findings indicate that acetylation/deacetylation of IDRs regulates LLPS and MLOs formation in response to stress conditions. Acetylome analysis uncovered the RNA helicase DDX3X, an essential SG constituent, as a new target for the deacetylase HDAC6. The acetylation of DDX3X impedes the generation of liquid droplets.^49^

##### Methylation

Methylation, which involves adding methyl (CH3) groups to arginine and lysine, stands out as a modification that alters biomolecular interactions. Unlike phosphorylation, methylation doesn’t add charge but increases the size and hydrophobicity of amino acids and changes the distribution of charge. Arginine methylation is prevalent among RNPs, influencing SG assembly through interactions with LCR regions.

In a subset of individuals with FTD, the presence of cytoplasmic FUS aggregates serves as a disease hallmark. Phase separation is inhibited by arginine methylation, which is missing in FTD-FUS patients.^312^ Loss of arginine methylation results in accumulation and aggregation of FUS in SG.^312^ In vitro studies demonstrate that FUS experiences concentration-dependent, reversible LLPS. These liquid FUS structures eventually transition into solid, fibrous aggregates over time, known as liquid-to-solid phase transition. Dimethylation of arginine residues on FUS diminishes LLPS and its association with SG, suggesting that the absence of arginine methylation, observed in FTD-FUS patients, contributes directly to FUS aggregation and pathology.^312^

The widely prevalent RNA modification, adenosine N6 methylation (m6A), affects RNA stability, transport, and translation. It has been demonstrated that the composition of the phase-separated transcriptome can be influenced by the number and distribution of m6A sites in mRNAs. This highlights the governance of cellular characteristics of m6A-modified mRNAs by LLPS principles.^313^ TDP43 has been identified as a recognizer of m6A RNA, with extensive RNA hypermethylation playing a crucial role in both TDP43 binding and self-regulation. Extensive hypermethylation of RNA observed in ALS spinal cord aligns with methylated TDP43 substrates, unveiling RNA modification targeting as an emerging avenue to regulate pathological protein phase transitions.^314^ Importantly, the investigator showed that TDP43-related neurotoxicity can be alleviated by knocking out the m6A reader YTHDF2,^314^ opening a potential new avenue for treating ALS.

##### PARylation (poly-ADP-ribosylation)

PARylation is a PTM where ADP-ribose molecules are incorporated into proteins by poly(ADP-ribose) (PAR) polymerases (PARPs). Recognizing PARylated proteins in MLOs is crucial for comprehending the impact of PARylation on MLOs and exploring its therapeutic potential.

FUS has been identified as a novel participant in the DNA damage response. FUS swiftly localizes DNA double-strand breaks, relying on PARPs activity. The interaction between FUS and PAR is mediated by arginine/glycine-rich domains, highlighting their direct connection.^315^ Notably, elevated PAR levels at DNA damage sites induce phase separation, leading to disease-associated protein aggregation.^316^ These findings might explain the mechanisms behind the therapeutic effects of PARP inhibitor in treating cancer^317^ and PD.^318^

Increased nuclear PARP-1/2 activity was found within ALS spinal cord motor neurons. Veliparib inhibited the generation of cytoplasmic TDP-43 aggregates by reducing nuclear PARP-1/2 activity. These findings suggest that PARP-1/2 inhibitors may have therapeutic potential for treating ALS.^319^ In the context of *Drosophila*, PARP inhibitors were shown to prevent TDP-43 accumulation and alleviate neurodegeneration.^320^ Furthermore, PARP silencing reduces neurotoxicity by preventing the condensate formation of hnRNPA1 and TDP-43 in a *Drosophila* model of ALS.^321^

### Regulation by small molecules

Amidst the quest for ALS therapeutics, the concept of ‘druggable’ condensates emerges as a tempting prospect.^258,322–329^ Approved drugs like Cisplatin and Mitoxantrone exhibit a tendency for partitioning into transcriptional condensates in tumor cells, opening avenues for exploration (Table 6).^330^ Compounds such as daunorubicin, pyrvinium, and pararosaniline, identified through high-content cellular screening, exhibit the potential to modulate condensate behaviors selectively. These compounds were able to prevent the accumulation of TDP-43, FUS, and HNRNPA2B1 in SGs and ameliorate neurotoxicity in ALS patients.^331^ Additionally, specific compounds have been pinpointed for their role in reducing stress-induced TDP-43 aggregation in PC12 cells, marking significant progress in the pursuit of effective ALS treatments.^332^

**Table 6:** Drugs modulate LLPS in disease

Drug	Function	Mechanism	Reference
Adriamycin (doxorubicin)	Anticancer drug	Adriamycin induces conformational change of chromatin by triggering the phase transition of H1 and forming fibrous aggregates	^326^
AR (Androgen Receptor)	AR antagonist	ET516 inhibits the transcriptional activity of AR by disrupting AR condensates, thereby inhibiting the proliferation and tumor growth of prostate cancer cells expressing AR-resistant mutant.	^328^
Cisplatin and Mitoxantrone	Antineoplastic drugs	The antineoplastic drugs are concentrated in specific protein condensates in tumor cells. The therapeutic efficacy of the drugs is related to their ability to partition into the condensate that harbors their target.	^330^
HIV	Anti-HIV drug	LLPS of SRC-1 is required for activating YAP oncogenic transcription. EVG inhibits YAP transcription by disrupting the LLPS of SRC-1.	^327^
SHP099	Protein tyrosine phosphatase (PTP)SHP2 inhibitor	LLPS serves as a gain-of-function mechanism involved in the pathogenesis of SHP2-associated diseases. SHP2 allosteric inhibitors attenuate LLPS of SHP2 mutants.	^329^

The interactions between TDP-43 and RNA drive the formation of abnormal inclusions, culminating in neurotoxicity. However, a promising therapeutic avenue unfolds as oligonucleotides, comprising TDP-43 target sequences, demonstrate their ability to prevent inclusions and neurotoxicity. This approach unveils the potential of oligonucleotides as a ray of hope in mitigating the neurotoxic impact of TDP-43 aberrations, providing a novel and targeted strategy in ALS treatment.

## Progress of targeting LLPS in clinic

### Neurodegenerative diseases

The aggregation of α-Syn is associated with the development of Parkinson’s disease. Research using in vitro reconstitution and cellular models has demonstrated that the LLPS of α-Syn occurs before its aggregation.^216^ Therefore, direct targeting of α-Syn has become a potential therapeutic strategy for Parkinson’s disease. Minzasolmin (UCB0599) is a small molecule targeting α-Syn misfolding and is currently being investigated as a potential treatment for Parkinson’s disease.^333^ Using the Line 61 transgenic mouse model of Parkinson’s disease, Price et al. found that minzasolmin-induced reductions in α-Syn pathology were associated with improvements in gait and decreased inflammatory markers.^333^ These findings provide evidence for determining appropriate human doses (ClinicalTrials.gov ID: NCT04658186; EudraCT Number 2020-003265).

The accumulation of over-phosphorylated, tangled microtubule-associated protein tau (MAPT) is a defining feature of AD.^334–341^ An exciting recent discovery is that tau has a strong tendency to undergo LLPS.^211^ A randomized phase 1b clinical trial investigated the effect of BIIB080, an antisense oligonucleotide targeting MAPT, on tau synthesis in patients with mild AD. The trial reported that BIIB080 was well tolerated and resulted in a dose-dependent decrease in total tau (t-tau) and phosphorylated tau (p-tau181) levels within the cerebrospinal fluid. Tau PET imaging showed reduced tau accumulation compared to placebo at week 25.^342^

The misfolding and mutation of Cu/Zn superoxide dismutase (SOD1) are frequently linked to ALS. SOD1 can accumulate within stress granules via LLPS.^343^ In a clinical trial involving 50 ALS patients with SOD1 mutations, CSF SOD1 concentrations decreased following 12 weeks of intrathecal administration of the antisense oligonucleotide tofersen at the highest dose.^344^

Huntington’s disease is caused by the expansion of a CAG trinucleotide repeat in the Huntingtin (HTT) gene.^345–347^ The HTT protein can assemble into liquid-like structures, which can transition into solid-like fibrillar assemblies within cells.^348^ This transition was further enhanced when the R200/205 methylation sites were modified.^349^ Therapeutic strategies aimed at lowering HTT levels are being developed to slow or halt the progression of Huntington’s disease.^350–355^ HTTRx, an antisense oligonucleotide developed to block HTT mRNA, reduces levels of mutant huntingtin protein. In patients with early Huntington’s disease, intrathecal administration of HTTRx did not result in serious adverse events and led to dose-dependent reductions in mutant huntingtin levels.^356^

The expansions of GGGGCC hexanucleotide repeat in C9ORF72 are the most common genetic cause of ALS and FTD.^223,357–362^ The hexanucleotide repeat causes a gain of toxicity to the neurons through phase separation.^363,364^ A one-time injection of antisense oligonucleotides targeting RNAs containing the repeat resulted in prolonged reductions in RNA foci and dipeptide-repeat proteins with improvements in behavioral deficits.^365^

### Cancer

The impact of genes related to LLPS on melanoma prognosis was investigated using the DrLLPS database, identifying TROAP as a gene marker associated with poor prognosis.^366^ Additionally, using The Cancer Genome Atlas and PhaSepDB datasets, Lai et al. identified five LLPS-related genes (LRGs) (BMX, FYN, KPNA2, PFKFB4, and SPP1) linked to the overall survival of hepatocellular carcinoma patients.^367^

LRGs extracted from PhaSepDB have proven valuable in predicting anticancer drug sensitivity in prostate cancer patients.^368^ FUS showed efficacy in predicting sensitivity to Nelarabine, Hydroxyurea, and Pemetrexed. USH1C emerged as a reliable predictor for Fluorouracil, Arsenic trioxide, and Everolimus sensitivity. Additionally, TAZ demonstrated predictive capabilities for Cladribine, Nelarabine, and Fludarabine sensitivity, while TPX2 was also effective in predicting Nelarabine sensitivity.^368^

### Cardiovascular diseases

RBM20 is a splicing factor involved in cardiovascular development.^185^ RBM20 mutations cause the relocation of RBM20 from nuclear splicing speckles to cytoplasmic condensates, resulting in the sequestration of mRNA, polysomes, and cardiac cytoskeleton proteins.^187^ Wyles et al. investigated the impact of β-adrenergic stress on familial DCM using human-induced pluripotent stem cell (hiPSC)-derived cardiomyocytes (CMs) from a patient with RBM20-related DCM. Their findings revealed that RBM20-deficient familial DCM hiPSC-CMs were more susceptible to stress, a vulnerability that could be therapeutically reduced by the β-blocker carvedilol and the Ca^2+^ channel blocker verapamil.^369^ This study paves the way for the development of therapeutic strategies to modify the progression of DCM.

### Hematological disorders

WASP mutations cause Wiskott–Aldrich syndrome (WAS), characterized by eczema, thrombocytopenia, immune deficiency, and thrombocytopenia.^288^ WASP regulates RNA splicing through a phase-separation process, and mutations in the WASP gene result in abnormal RNA splicing.^289^ Gene therapy using modified autologous CD34^+^ cells is an emerging treatment for WAS. Ferrua et al. presented safety and efficacy data from an interim analysis of a phase 1/2 clinical study on patients with severe WAS who underwent lentiviral vector-based gene therapy.^370^ The treatment involved a single intravenous infusion of autologous CD34^+^ cells genetically modified with a lentiviral vector encoding human WAS cDNA. The overall survival rate was 100%, with successful and sustained engraftment of genetically corrected HSPCs in all patients. The study also demonstrated improved immune function, as indicated by normalized in vitro T-cell function and the discontinuation of immunoglobulin supplementation in seven patients with follow-up longer than one year. Additionally, severe infections decreased, and platelet counts significantly improved. These findings were corroborated by a recent phase 1/2 clinical trial, which reported outcomes for five patients with severe WAS who received gene therapy using a self-inactivating lentiviral vector expressing human WAS cDNA.^371^ All patients were alive and in good health, with sustained multilineage vector gene marking. Universal clinical improvements were observed in eczema, infections, and bleeding diathesis. Notably, the most significant enhancements in platelet count and cytoskeletal function in myeloid cells occurred in patients with higher vector copy numbers in the transduced product.

## Conclusions and perspective

The exploration into MLOs has revealed a dynamic aspect of cellular organization, challenging traditional compartmentalization and adding depth to our comprehension of cellular dynamics. This extensive analysis delves into the varied functions of MLOs, encompassing SGs, P-bodies, the nucleolus, and the principles of LLPS across diverse biological scenarios. As we wrap up this investigation, we contemplate the current state of knowledge and offer insights into the future directions of this rapidly evolving field.

The identification and characterization of MLOs have unveiled the intricate nature of cellular organization. These formations, facilitated by LLPS, exemplify the adaptable and responsive characteristics of cellular constituents. The complex interaction between membrane-bound and MLOs highlights the precise coordination governing cellular activities. MLOs assume diverse roles in cellular mechanisms like mRNA regulation, protein equilibrium, and stress response, underscoring their significance in preserving cellular equilibrium.

As research progresses, the influence of MLOs expands beyond traditional cellular mechanisms. Their significance is apparent in diverse biological contexts, from regulating gene expression in cancer cells to coordinating metabolism and cardiac function and impacting the dynamics of aging and neurodegenerative diseases. This broadening biological scope underscores the ubiquity and versatility of MLOs, positioning them as central actors in cellular physiology and pathology.

The therapeutic potential of MLOs is an emerging area of exploration. Targeting these dynamic structures provides a novel avenue to intervene in cellular processes associated with various ailments. Small molecules that modulate LLPS or influence the dynamics of specific MLOs hold promise as prospective therapeutics. Ongoing endeavors to translate knowledge into therapeutic tactics underscore the potential impact of this field on future medical interventions.

As therapeutic strategies targeting MLOs progress, ethical considerations must remain paramount. Understanding potential off-target effects and unintended consequences of modulating LLPS or MLOs dynamics is critical. Ethical frameworks should guide the development and implementation of therapeutic interventions to ensure safety and efficacy.

Despite significant progress in understanding MLOs, challenges persist in comprehending their assembly, dynamics, and functionalities. The ephemeral and dynamic nature of these structures presents experimental hurdles, necessitating innovative approaches to observe and manipulate MLOs within living cells. Further inquiry is essential to decipher the principles dictating LLPS behavior and the factors influencing condensate formation.

To fully elucidate the complexities of MLOs, a multidisciplinary approach and reagents (Table 7) are imperative.^372^ Integration of techniques from cell biology, biophysics, biochemistry, and computational biology is essential to attain a comprehensive understanding of MLOs formation, regulation, and functionalities. Collaborative endeavors across disciplines will drive innovation, enabling researchers to effectively address the intricacies of these dynamic structures.

**Table 7:** Reagents modulate LLPS

Reagent	Function	Mechanism	Reference
Dextran	Crowding reagent	Crowding reagents to promote the formation of MLO	^3^
Ficoll	Crowding reagent	Crowding reagents to promote the formation of MLO	^3^
1,6-Hexanediol (1,6-HD)	1,6-HD is an inhibitor and indicator of phase separation	1,6-HD disrupts LLPS by interfering the weak hydrophobic protein-protein or protein-RNA interactions.	^3^
Lipoamide and lipoic acid	Inhibit stress granule formation	Dissolve stress granule by affecting the kinetics of phase separation	^374^
Poly(ethylene glycol)	Crowding reagent	Crowding reagents to promote MLO	^3^

The field of MLOs stands on the brink of ongoing expansion and exploration. Future investigations may focus on elucidating the functions of specific proteins, RNA molecules, and PTMs in governing MLOs. Given the evolutionary connection between PTM and LLPS, it is likely that additional types of PTMs remain to be discovered. For instance, the strong electronic neutralization effect of O-glycosylation may inhibit potential LLPS. Investigating how PTMs regulate LLPS will undoubtedly become a key focus of future research.^373^ The potential of lysosome-associated condensates in therapeutic applications should be further explored. For instance, Aloperine (ALO), a natural alkaloid, has demonstrated the ability to inhibit tumor growth by directly targeting lysosomes in glioma cells.^374^ Progressing technologies, including advanced imaging methodologies and CRISPR-based techniques, are expected to bolster our capacity to manipulate and scrutinize these structures in real time.

In summary, MLOs serve as connectors between cellular compartments, challenging traditional perceptions of cellular organization. Their dynamic essence, illuminated through LLPS, underscores the inherent flexibility within cellular processes. The convergence of technological advancement, interdisciplinary cooperation, and ethical considerations will steer us toward unlocking the complete potential of these dynamic structures in both health and disease. The pursuit of comprehending MLOs persists, promising a future where their contributions to cellular dynamics are fully understood and leveraged for therapeutic progress.

### Unanswered questions

While significant advancements have been achieved in understanding MLOs and their roles in cellular function, several unanswered questions persist, particularly regarding their involvement in human diseases. Here are some of the key unanswered questions in this field:

#### Disease-specific mechanisms

What are the specific molecular mechanisms underlying the dysregulation of MLOs in different human diseases? While it is known that disruptions in MLOs play a role in disease development, the precise molecular events and interactions involved in this process remain incompletely understood.

#### Contributions to disease progression

How do alterations in MLOs contribute to the progression of various human diseases? While some studies have linked MLOs dysfunction to disease initiation, it is unclear how these alterations evolve over time and exacerbate disease severity.

#### Cell-type specificity

Are MLOs present in all cell types, or are they restricted to particular cell types? Do MLOs dysfunctions exhibit cell-type specificity in the context of human diseases? Different cell types may have distinct MLOs compositions and functions, and understanding how MLOs dysregulation affects specific cell types could provide insights into disease heterogeneity.

#### Interplay with genetic and environmental factors

How do genetic and environmental factors interact with MLOs dysregulation to influence disease susceptibility and progression? When might the reprogramming of MLOs occur? Can MLOs be passed down to daughter cells during cell division? Genetic variants and environmental stressors may modulate MLOs function and contribute to disease risk, but the underlying mechanisms are not fully elucidated.

#### Diagnostic and prognostic markers

Can MLOs dysregulation serve as diagnostic or prognostic indicators for human diseases? Identifying specific MLOs alterations associated with disease states could facilitate the advancement of biomarkers for early disease detection and prognostication.

#### Concept issue

The concept of LLPS is somewhat inaccurate, as there is also a gel state and solid state. Therefore, whether it should be changed to “phase separation” or “phase transition” is debatable. The intrinsically disordered region (IDR) is important for LLPS, but protein phase transition doesn’t necessarily require an IDR. Proteins with disordered regions don’t necessarily undergo phase transition. Therefore, the mechanisms underlying the formation of LLPS remain to be explored.

#### The interaction and location

The interactions between MLOs and membrane-bound organelles under normal and abnormal conditions require further research. The half-life and number of MLOs within a particular type of cells need further clarification. Previous studies focus on individual molecule, their upstream and downstream regulation. MLOs concentrate key molecules together, acting in specific times and spaces (nucleus, cytoplasm, potential mitochondria). However, the dynamic network mediated by MLOs remains largely unexplored.

Addressing these unanswered questions will require interdisciplinary approaches combining genetics, molecular biology, cell biology, biochemistry, and clinical research. By elucidating the complex roles of MLOs in human diseases, researchers will uncover novel therapeutic targets and diagnostic tools to improve patient outcomes.
